# Distribution and prognostic impact of microglia/macrophage subpopulations in gliomas

**DOI:** 10.1111/bpa.12690

**Published:** 2019-01-15

**Authors:** Pia S. Zeiner, Corinna Preusse, Anna Golebiewska, Jenny Zinke, Ane Iriondo, Arnaud Muller, Tony Kaoma, Katharina Filipski, Monika Müller‐Eschner, Simon Bernatz, Anna‐Eva Blank, Peter Baumgarten, Elena Ilina, Anne Grote, Martin L. Hansmann, Marcel A. Verhoff, Kea Franz, Friedrich Feuerhake, Joachim P. Steinbach, Jörg Wischhusen, Werner Stenzel, Simone P. Niclou, Patrick N. Harter, Michel Mittelbronn

**Affiliations:** ^1^ Edinger Institute, Institute of Neurology Goethe University Frankfurt Frankfurt am Main Germany; ^2^ Department of Neurology Goethe University Frankfurt Frankfurt am Main Germany; ^3^ Dr. Senckenberg Institute of Neurooncology Goethe University Frankfurt Frankfurt am Main Germany; ^4^ Department of Neuropathology Charité Berlin Berlin Germany; ^5^ NORLUX Neuro‐Oncology Laboratory, Department of Oncology Luxembourg Institute of Health (LIH) Luxembourg; ^6^ Department of Oncology Luxembourg Institute of Health (LIH) Luxembourg; ^7^ German Cancer Consortium (DKTK) Heidelberg Germany; ^8^ German Cancer Research Center (DKFZ) Heidelberg Germany; ^9^ Institute of Neuroradiology Goethe University Frankfurt Frankfurt am Main Germany; ^10^ Department of Neurosurgery Goethe University Frankfurt Frankfurt am Main Germany; ^11^ Luxembourg Centre of Neuropathology (LCNP) Luxembourg; ^12^ Institute of Pathology and Neuropathology Medical University Hannover Hannover Germany; ^13^ Senckenberg Institute of Pathology Goethe University Frankfurt Frankfurt am Main Germany; ^14^ Institute of Legal Medicine Goethe University Frankfurt Frankfurt am Main Germany; ^15^ Institute of Neuropathology, University Clinic Freiburg Freiburg Germany; ^16^ Department of Gynecology University of Wuerzburg Wuerzburg Germany; ^17^ KG Jebsen Brain Tumour Research Center, Department of Biomedicine University of Bergen Bergen Norway; ^18^ Luxembourg Centre for Systems Biomedicine (LCSB) University of Luxembourg Luxembourg; ^19^ Laboratoire national de santé (LNS) Dudelange Luxembourg

**Keywords:** glioma, glioma‐associated microglia and macrophages, immune polarization, tumor microenvironment

## Abstract

While the central nervous system is considered an immunoprivileged site and brain tumors display immunosuppressive features, both innate and adaptive immune responses affect glioblastoma (GBM) growth and treatment resistance. However, the impact of the major immune cell population in gliomas, represented by glioma‐associated microglia/macrophages (GAMs), on patients’ clinical course is still unclear. Thus, we aimed at assessing the immunohistochemical expression of selected microglia and macrophage markers in 344 gliomas (including gliomas from WHO grade I–IV). Furthermore, we analyzed a cohort of 241 IDH1R132H‐non‐mutant GBM patients for association of GAM subtypes and patient overall survival. Phenotypical properties of GAMs, isolated from high‐grade astrocytomas by CD11b‐based magnetic cell sorting, were analyzed by immunocytochemistry, mRNA microarray, qRT‐PCR and bioinformatic analyses. A higher amount of CD68‐, CD163‐ and CD206‐positive GAMs in the vital tumor core was associated with beneficial patient survival. The mRNA expression profile of GAMs displayed an upregulation of factors that are considered as pro‐inflammatory M1 (eg, *CCL2, CCL3L3, CCL4, PTGS2*) and anti‐inflammatory M2 polarization markers (eg, *MRC1, LGMN, CD163, IL10, MSR1*), the latter rather being associated with phagocytic functions in the GBM microenvironment. In summary, we present evidence that human GBMs contain mixed M1/M2‐like polarized GAMs and that the levels of different GAM subpopulations in the tumor core are positively associated with overall survival of patients with IDH1R132H‐non‐mutant GBMs.

## Introduction

The tumor microenvironment substantially influences tumor progression and first approaches targeting the immunological tumor microenvironment entered clinical trials 13, 18. Especially cytotoxic tumor infiltrating lymphocytes (TILs) might be crucial and are mostly associated with improved survival in peripheral neoplasms 11. In high‐grade gliomas, that still display a dismal prognosis 48, there is controversial data about the prognostic relevance of TILs 2, 17, 21, 24, 26. *In vitro* experiments have shown promising antitumoral effects of TILs and NK cells 33. However, the majority of immune cells in gliomas are not TILs but rather glioma‐associated microglia and macrophages (GAMs, referring explicitly to microglia and macrophages in the glioma microenvironment in distinction to microglia and macrophages (M/M) without a relation to any tumor microenvironment) 14, 34. The origin of GAMs was investigated in several different GBM mouse models showing discrepant findings regarding the amount of recruited macrophages and microglia that contribute to the total GAM population 44. GAMs infiltrate the tumor center via chemoattraction 38, potentially playing a role in TIL recruitment. Nevertheless, the functions and the ability to initiate an effective antitumor immune response as well as the prognostic impact onto patients’ clinical course of tumor‐associated macrophages (TAMs) still remain unclear and are ambivalently discussed (eg, positive 10, 37 vs. negative 39, 51 prognostic effects). This has recently been reviewed in detail with regard to GAMs 15, 23, 41, 44. External stimuli like interferon‐γ (IFN‐γ), tumor necrosis factor (TNFα) or the interleukins IL‐4 and IL‐10 considerably influence macrophages in general 28 and tumor‐associated macrophages (TAMs, referring to macrophages in non‐CNS cancer entities) eg, 5. To classify macrophage properties and functions, the concept of M1/M2 immune polarization was introduced 32. TAMs are considered to develop a so‐called M2 phenotype with anti‐inflammatory functions in most peripheral high‐grade tumors 46, being associated with tissue remodeling, angiogenesis that might contribute to tumor progression 6, 28 and poor prognosis eg, 5. However, the M1/M2 model is strongly debated in general 14 and evidence for its suitability for neoplasms of the central nervous system (CNS) is still poor 20, 49. Despite potentially distinct immunological properties of macrophages and microglia, it is still difficult to differentiate the exact origin of GAMs 9, 43, 44.

Thus, the aim of our study was to characterize distinct GAM subpopulations in the microenvironment of astrocytomas and to assess the immunological properties of GAMs in high‐grade astrocytomas. We investigated the prognostic impact of GAM subpopulations in a cohort of 241 patients with IDH1R132H‐non‐mutant glioblastoma (GBM) and additionally in GBM patient‐derived orthotopic xenografts (PDOX) models to evaluate a potential rationale for GAM‐targeting or immune polarization associated therapy approaches 16, 25, 27.

## Material and Methods

### Patient tissue and tissue microarrays

We investigated tissue micro arrays (TMA) containing paraffin embedded samples from 344 patients. The histopathological diagnoses were performed by board certified neuropathologists (MM, PNH). Diagnosis of tumor samples was retrospectively adapted according to the WHO criteria of 2016 including immunohistochemical analysis of the IDH1R132H‐mutation status (IDH1R132H‐immunohistochemistry (IHC) positive in IDH1R132H‐mutant and negative in IDH1R132H‐non‐mutant gliomas). We included 44 pilocytic astrocytomas WHO grade I (all IDH1R132H‐non‐mutant), 14 diffuse astrocytomas WHO grade II (8 IDH1R132H‐mutant, 6 IDH1R132H‐non‐mutant), 35 anaplastic astrocytomas WHO grade III (19 IDH1R132H‐mutant, 16 IDH1R132H‐non‐mutant) and 251 GBMs WHO grade IV (10 IDH1R132H‐mutant, 241 IDH1R132H‐non‐mutant). We only analyzed treatment‐naive patients at first diagnosis; recurrent glioma patients were excluded. During preparation of TMAs (previous descriptions of the cohort and TMA preparation 1), we carefully selected representative tissue samples on whole mount sections consisting of vital tumor regions and avoiding marked necrotic areas. To rule out intraindividual differences, up to three repeated cores of the same patients were included in the TMAs. The first core of each patient was used for statistical analyses to avoid subjective bias. We additionally included and separately analyzed infiltration zones (IZ, n = 39, distinct GBM area with 50%–70% reduction of cell density as compared to the corresponding vital tumor tissue; both areas had previously been identified in whole mount sections) and normal‐appearing gray (NAGM, n = 62) or white matter (NAWM, n = 19) from GBM samples that were analyzed separately. As we investigated diffusely infiltrating tumors, which invade gray as well as white matter, we combined NAGM and NAWM to normal appearing brain tissue (NAB, n = 81). Normal human autopsy brain tissues and tonsil tissue were derived from the tissue bank of the Edinger Institute (Neurological Institute), Frankfurt, Germany. Tumor samples were derived from the local Biobank UCT Frankfurt, Germany. The usage of human patient material was approved by the local ethical committee (GS‐04/09, GS‐249/11, SNO‐01‐2015).

### Whole DNA methylome analyses

Exemplarily, we investigated a cohort of 11 patients with DNA‐methylation‐based classification of IDH‐wild‐type GBMs 7 using the EPIC 850k whole methylome Chip (Illumina, San Diego, CA, USA) following standard protocols for tissue and DNA processing. Hybridization was performed as indicated by the manufacturer. Data were preprocessed using Illumina Genome Studio. Further analysis was performed using JMP 14.0 (SAS, Cary, NC, USA). Of these 11 DNA‐methylation‐based IDH‐wild‐type GBMs, 2 patients were previously diagnosed as IDH1R132H‐non‐mutant anaplastic astrocytomas WHO grade III and one patient was diagnosed as IDH1R132H‐non‐mutant diffuse astrocytoma WHO grade II based on histopathological evaluation. In these patients, neuroradiological signs of necroses and contrast enhancement indicating the morphologically defined GBM microenvironment were likewise not present as evaluated by a board certified neuroradiologist (MME). Thus, these three cases were described as discordant to the DNA‐methylome analysis. We compared the amount of Iba1‐positive GAMs of these three patients with those of the remaining eight patients with concordant histopathological and DNA‐methylation‐based classification of IDH‐wild‐type GBM.

### Patient‐derived orthotopic xenografts

GBM biopsies were collected at the Neurosurgical Department of the Centre Hospitalier in Luxembourg and of Haukeland University Hospital, in Bergen, Norway from patients who have given their informed consent. The use of patient material has been approved by the local ethics committees [National Ethics Committee for Research of Luxembourg (CNER); Project number: REC‐LRNO‐20110708], local ethics committee Haukeland University Hospital, Bergen). Multicellular organotypic GBM spheroids containing heterogeneous cell populations and GBM brain tumor‐derived initiating cells (BTICs) were prepared as previously described and implanted in immunodeficient mice 3. Briefly, mechanically minced samples were seeded on agar coated flasks (0.85%) and allowed to form spheroids for 2 weeks at 37°C under 5% CO2 and atmospheric oxygen in DMEM medium, 10% FBS, 2 mM L‐Glutamine, 0.4 mM nonessential amino acids (NEAA) and 100U/mL Pen‐Strep (all from Lonza). BTICs (spheroids of 300–400 µm diameter, 6 per animal) were implanted into the brain of NOD/SCID mice 36. Mice were anesthetized with a mixture of ketamine (100 mg/kg) and xylazine (10 mg/kg) and fixed in a stereotactic frame (Narishige Group, Tokyo, Japan), and a small hole was drilled in the skull. BTICs were slowly injected through a Hamilton syringe (Hamilton, Reno, NV, USA) into the right frontal cortex. The animals were sacrificed upon severe neurological or behavioral abnormalities. The handling of the animals and the surgical procedures were performed in accordance with the European Directive on animal experimentation (2010/63/EU) and the Luxembourgish law. Twenty‐six mice were analyzed in the study carrying xenografts derived from 12 GBM patient tumors (1–3 mice per PDOX model). The local ethical committee for animal welfare approved the protocols.

### Immunohistochemistry

For IHC analyses, the following antibodies were used: anti‐leukocyte common antigen CD45 (Dako, Hamburg, Germany; M0701, dilution for IHC 1:2000), anti‐CD68 (Dako; M0876, dilution for IHC 1:200), anti‐CD163 (Leica Biosystems, Novocastra, Nußloch, Germany; NCL‐CD163, dilution for IHC 1:50), anti‐CD206 (abcam; ab117644, dilution for IHC 1:100), anti‐Iba1 (Wako, Neuss, Germany; 019‐19741, dilution for IHC 1:1000) and anti‐MHCII (Dako; M0775, dilution for IHC 1:1000). IHC were performed according to standardized protocols using Discovery XT automated immunostainer (Ventana Medical Systems, München, Germany) as previously published 1 and analyzed using a light microscope (BX41, Olympus, Hamburg, Germany).

### Manual quantification of immunohistochemistry and statistical analysis

IHC of different M/M markers (CD68, CD163, CD206, Iba1) was evaluated as the percentage of positive cells related to the total cell number by visual estimation of the entire tissue cores of patients in TMAs. Intravascular cells were excluded by morphology (evaluated by board certified neuropathologists). TMA cores with mainly necrotic tissue (necrotic area >30%) were excluded from the statistical analysis of the TMAs. In TMA cores with <30% of necrosis, necrotic parts were excluded from GAM quantification. Since some of the aforementioned M/M markers might in fact predominantly but not exclusively be expressed by GAMs in the total GBM microenvironment, we additionally applied suggestive histomorphologic criteria for quantification that together with the respective IHC markers did most likely reflect CD163‐, CD206‐ or CD68‐ positive GAMs. We performed correlation analysis based on Spearmans *ρ* testing of repeated TMA cores of the same patients to rule out intraindividual differences. Since homoscedasticity and normal distribution were not given for all analyses, nonparametric Wilcoxon’s test with subsequent adjustment of the *P*‐values by the Bonferroni–Holm method was used. *P*‐values were indicated including their 95% confidence intervals (*P ≤ 0.05; **P ≤ 0.01; ***P ≤ 0.001). To exclude a potential inter‐ or intraobserver variation of manual IHC quantification, we correlated (Spearmans *ρ* testing) the analyses of two independent raters and the analyses of one rater performed at two different time points.

Additionally, the amount of Iba‐1 positive GAMs was manually quantified in a cohort of DNA‐methylation‐based classified IDH‐wild‐type GBMs with partly (n = 3) discordant results between histopathological grading and DNA‐methylation profiling. Statistical analysis was performed using GraphPad Prism 7 software.

Further, manual quantification of Iba1‐positive cells of whole mount tissue samples of PDOX models was performed. Vital tumor core was analyzed separately from infiltration zone (IZ) and normal appearing brain tissue (NAB). The association of patient (IDH1R132H‐non‐mutant GBMs) and murine survival with response variables was assessed by Kaplan–Meier analyses as previously described using a median split for all M/M markers 53 and additionally a best split for Iba1‐expression. Statistical analysis was performed using JMP 14.0 software (SAS). A significance level of alpha = 0.05 was chosen for all testings.

### Automated quantification of immunohistochemistry and statistical analysis

In a second approach, we controlled accuracy and precision of the above‐mentioned manual analysis with automated image analysis, a threshold‐based assessment of stained area, implemented in MATLAB (MathWorks, Aachen, Germany) exemplarily for Iba1‐IHC staining in our TMA patient cohort. The thresholds were based on minimum entropy estimation, applied on the DAB and hematoxylin channels after color deconvolution. For the DAB channel, the threshold was estimated in two steps. First, a threshold was estimated from the whole spot. Second, the final threshold was estimated from the areas found with the first threshold. This procedure is more robust against varying background staining than applying a single threshold. The estimation was done for each TMA spot individually. The resulting ratio of DAB area to total area was correlated with the manual quantification of Iba1‐IHC.

### MACS® isolation of human microglia and macrophages

GAMs were isolated by CD11b‐based magnetic cell sorting (MACS®) from in total n = 9 native human high‐grade astrocytomas (n = 7 IDH1R132H‐non‐mutant GBMs, n = 1 IDH1R132H‐mutant GBM, n = 1 IDH1R132H‐mutant anaplastic astrocytoma, WHO grade III) and primary microglia from n = 3 normal brain autopsy cases with a short post‐mortem delay as described previously 53. Isolated cells were characterized by FACS reaching a purity of >95% 53 and by Iba1, MHCII and CD45 immunocytochemistry. Tissue samples for M/M isolation were obtained from the departments of neurosurgery, pathology and legal medicine of the Goethe University, Frankfurt, Germany. The use of human tumor and control material was approved by the local ethical committee (GS‐04/09, GS‐249/11, SNO‐01‐2015).

### Cell culture of primary human tumor cells

Primary cultures from native human GBM specimens were established and grown in DMEM/10% FCS or neurosphere medium [50 mL DMEM‐F12 (Gibco Invitrogen) supplemented with 50 μL epidermal growth factor (EGF, PeproTech, Hamburg, Germany), 100‐μL basic fibroblast growth factor (bFGF, PeproTech), 1 mL B27 Supplement (Gibco Invitrogen), 0.5 mL N‐2‐ hydroxyethylpiperazine‐N′‐2‐ethanesulfonic acid (HEPES, Gibco Invitrogen), 0.5 mL P/S (Sigma Aldrich)] as described previously 53.

### RNA microarray and genome wide gene expression analysis

RNA microarray analyses were performed using isolated microglia from normal white matter (WM) of n = 3 patients and GAMs from n = 6 native human GBM patients (n = 5 IDH1R132H‐non‐mutant, n = 1 IDH1R132H‐mutant). Microglia from WM were pooled for the RNA microarray caused by lower RNA amounts. Total RNA was extracted from MACS® isolated cells as described previously 53. Only RNAs found free of contamination as determined by spectrophotometry using the NanoDrop® ND‐1000 system were further analyzed. RNA analysis using Bioanalyzer 2100 and RNA pico chips (Agilent Technologies, Diegem, Belgium) revealed that all samples were partially degraded with RIN ranging from 3.5 to 7.10. RNAs were further processed using the Affymetrix sensationPlus FFPE amplification and whole‐transcriptome labeling kit as recommended by the manufacturer (protocol P/N 703089 Rev. 3). Briefly, 50 ng of input RNAs were linearly amplified and biotin‐labeled, and the resulting end‐labeled cDNAs (5 µg) were hybridized onto the Affymetrix GeneChip Human gene 2.0 ST arrays for 17 h. Arrays were then washed and stained using the Affymetrix GeneChip WT Terminal Labeling and Hybridization kit, before being scanned using a GeneChip Scanner 3000 following the manufacturer’s instructions (user manuals number PN 702731 & P/N 702569). CEL files generated after upon array scanning were imported into the Partek Genomics SuiteTM (GS) 6.6 for further analysis. Partek options were set up for GC‐content adjustment, robust multi‐array (RMA) background correction, quantile normalization, log2 transformation and mean summarization. Data were first preprocessed to estimate transcript cluster expression levels from raw probe signal intensities. Resulting expression data were then analyzed by R statistical environment (https://cran.r-project.org). The quality of the data was assessed through density plots, relative log expression signal (RLE) and Pearson’s correlation. Principal component analysis was used to identify potential source of variability in the dataset. Finally, differentially expressed genes (DEGs) between GAMs and normal WM microglia samples were determined. Genes found with an absolute Log2 fold change >= 2 were considered for further analysis. Pearson transformation was applied to analyze coexpression of genes.

### Bioinformatic analyses

The Ingenuity Pathway Analysis database (IPA) (Ingenuity Systems, Redwood City, CA, USA, www.ingenuity.com) and the DAVID® database were used for data mining, including functional analyses, upstream analysis, gene network reconstruction, as well as up‐ and downstream analysis of interaction networks. In DAVID®, biological functions deregulated between GAMs and the reference WM microglia were determined from the set DEGs considering enrichment scores >2. In IPA, the mapping of the DEGs resulted in a list of 956 analysis ready molecules that were further processed considering only the experimentally validated set of data referenced in the IPA database. Upstream regulators were deemed (i) significant if associated with a *P*‐value of overlap <10^2^; (ii) activated if associated *z*‐score was >2 or (iii) inhibited if *z*‐score was <−2.

### Quantitative real‐time polymerase chain reaction (qRT‐PCR)

The qRT‐PCR served to confirm differential expression of a subset of M/M polarization genes regulated in the RNA microarray in GAMs as compared to normal microglia. Extraction of total RNA and reverse transcription into cDNA from microglia from normal WM and from GAMs from native human high‐grade astrocytomas was performed as described previously 53. The quantitative PCR (qRT‐PCR) reactions were prepared in a final volume of 20 µL. The TaqMan Fast Universal PCR Master Mix, the target assay (Applied Biosystems) and 20 ng of cDNA were used for the analysis of the TaqMan primers *CCL22*, *IL10*, *MRC1 *(CD206), *NOS2 *(iNOS), *Arg1*. For SYBR (Applied Biosystems, Foster City, CA, USA) based qRT‐PCR of the primers *CCL2* (fw 5′‐GAAAGTCTCTGCCGCCCTTCT‐3′, rv 5′‐GGACACTTGCTGCTGGTGATT‐3′), *CD14* (fw 5′‐CACTTTCCAGCTTGCGCCTAC‐3′, rv 5′‐AAAGGCAGGCGAGTGTGCTTG‐3′), *CD163* (fw 5′‐GGACATGAGTCCCATCTTTCAC‐3′, rv 5′‐AGCTCCACTCTGCCCTCACAC‐3′), *CD163 Molecule Like 1* (*CD163L1)* (fw 5′‐GTTCTTGGAGCACCTCCCTGT‐3′, rv 5′‐GATCAAAGCACTGCCCTCTGG‐3′), *IL1B* (fw 5′‐TGAAGCTGATGGCCCTAAACAG‐3′, rv 5′‐TGCTGTAGTGGTGGTCGGAGA‐3′) *MRC1* (fw 5′‐GACTCCCGAACCCAAATGTCC‐3′, rv 5′‐TCGCCATATTGTTTGCTGTTCC‐3′) *MSR1* (fw 5′‐GGAGCAGTGGGATCACTTTCA‐3′, rv 5′‐CGAGGAGGTAAAGGGCAATCA‐3′), *PTGS2* (fw 5′‐TCCTCCTGTGCCTGATGATTG‐3′, rv 5′‐TGGCCCTCGCTTATGATCTGT‐3′) and *TLR2* (fw 5′‐CTCGGAGTTCTCCAGTGTTT‐3′, rv 5′‐CCAGTGCTTCAACCCACAAC‐3′) per triplicate 3.25 µL (approx. 150 ng) cDNA, 6.5 µL primers, and 32.5 µL SYBR in 22.75 µL sterile DNAse‐RNAse free water were applied. *RPLP0* (fw 5′‐GAGTCCTGGCCTTGTCTGTGG‐3′, rv 5′‐TCCGACTCTTCCTTGGCTTCA‐3′) and *18S* (fw 5′‐CTTTGGTCGCTCGCTCCTC‐3′, rv 5′‐CTGACCGGGTGGTTTTGAT‐3′) were used as internal standard controls. Primers were designed with the Primerblast tool. Primermix of forward (fw) and reverse (rv) primers (Sigma Aldrich, Steinheim, Germany) were prepared at a concentration of 100 mM. Amplicons were loaded onto ethidumbromide gels to exclude unspecific binding. Serial dilutions were used to generate standard curves for each gene. All analyses were performed in triplicate, and the dCT, ddCT and *R* value (% of WM control group) were determined.

### Immunoblotting

Protein lysates were generated by mechanical and enzymatic treatment of cryo‐conserved human brain tumors, primary tumor cells and M/M as described previously 53. The protein concentration was determined according to the manufacturer’s protocol of the Micro BCA™ Protein Assay Kit (Thermo Scientific, Dreieich, Germany). The electrophoretic separation of the denatured proteins was performed on 12.5% SDS‐polyacrylamide gels followed by a blotting process as described previously 53. Blots were blocked in 1x Roti‐Block blocking buffer (Roth, Karlsruhe, Germany) and then incubated with the primary antibodies Iba1 (Wako, 016‐20001, dilution for WB 1:1000) and beta‐actin (Abcam, ab8227, dilution for WB 1:2500) as a loading control. Immunodetection was performed by HRP enzyme‐coupled secondary antibodies, which oxidize luminol (Santa Cruz Biotechnology, Heidelberg, Germany) resulting in a chemoluminescent reaction on X‐ray films (Super RX, Fujifilm Europe GmbH, Düsseldorf, Germany).

## Results

### GAMs display a mixed M1/M2‐like polarization profile

**Figure 1:**
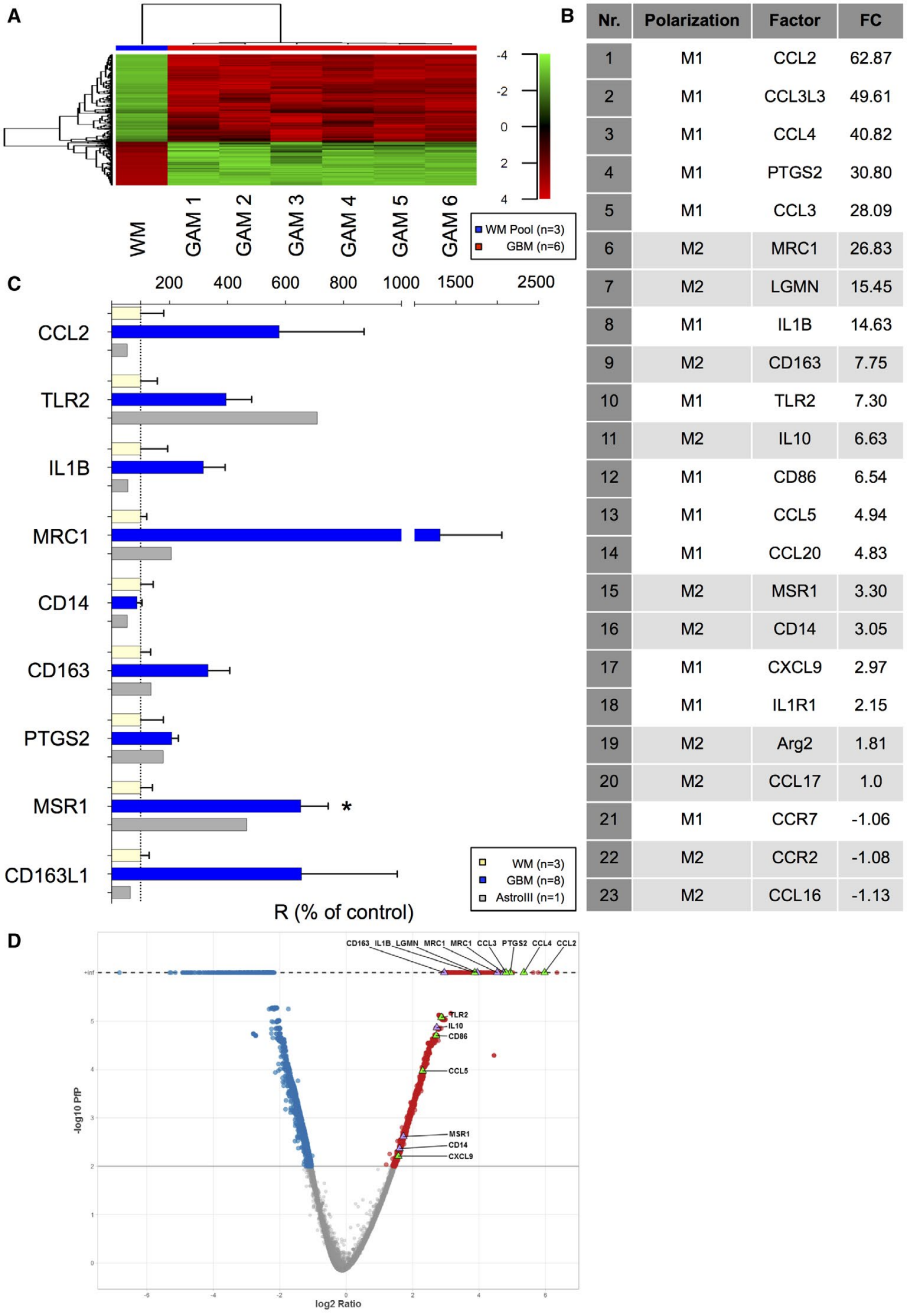
*GAMs display a mixed M1/M2 immune phenotype*. **A.** Heatmap showing the expression profiles of 979 genes that were differentially expressed between GAMs from 6 GBMs (n = 5 IDH1R132H‐non‐mutant, n = 1 IDH1R132H‐mutant) and reference microglia (from three pooled samples from normal WM) based on an absolute Log2 Fold‐change >= 2. The expression pattern was similar among the different GAM samples and clearly different from the reference. Hierarchical clustering was performed using the Ward’s minimum variance method. Red indicates a high raw intensity in the microarray, gradually decreasing, indicated in green. **B.** The most significantly regulated molecules of immune polarization in GAMs vs. WM microglia are depicted (FC: fold‐change GAM/WM). **C.** The mRNA expression profile of selected M/M polarization markers differentially expressed in the microarray (*CCL2, TLR2, IL1B, MRC1, CD14, CD163, PTGS2, MSR1, CD163L1*) was assessed via qRT‐PCR in GAMs of eight GBM patients (blue, n = 7 IDH1R132H‐non‐mutant, n = 1 IDH1R132H‐mutant) and one patient with IDH1R132H‐mutant anaplastic astrocytoma WHO grade III (gray) in comparison to the median expression of the respective genes in WM microglia (n = 3, yellow). **D.** In the Volcano plot, the pfp (−log10) vs. gene expression ratio (log2) of all features present on the microarray is shown. Blue and red correspond to significantly (pfp < 0.01) down or upregulated genes. Upregulated M1 or M2 genes are highlighted in green or purple triangles.

CD11b‐positive GAMs from fresh astrocytoma tissue and microglia from normal WM were sorted by MACS® (Figure S1A) reaching a high purity (Figure S1B to G and as previously demonstrated (Figure S2 of 53). Iba1 expression in protein lysates of purified GAMs, primary GBM cultures and whole tumor tissue lysates all correspondingly derived from the same respective patient confirmed the content of GAMs (Figure S1B). Transcriptome analysis identified 979 differentially expressed genes showing a very distinct expression pattern in GAMs of GBM patients in contrast to normal WM microglia samples (Figure 1A) that were used as reference for further analyses. Gene expression profiles were found relatively stable in the GAM samples (Figure 1A). Among the differentially expressed genes between GAMs and normal WM microglia, many were recognized as inflammatory molecules (Figure 1B–D) belonging to both M1‐like (eg,: *CCL2, CCL4, CCL3, IL1B, TLR2, CD86, CCL5 *and *PTGS2*) and M2‐like polarization markers (eg,: *MRC1, LGMN, IL10, MSR1, CD14, CD163*). Moreover, the latter might functionally be partly involved in phagocytic and adhesion processes in the GBM microenvironment as indicated by Ingenuity Pathway Analysis (IPA) (Table S1, S2). The validation qRT‐PCR of selected M/M polarization markers (*CCL2, TLR2, IL1B, MRC1, CD14, CD163, PTGS2, MSR1, CD163L*) in GAMs from eight GBM patients (n = 7 IDH1R132H‐non‐mutant, n = 1 IDH1R132H‐mutant) and one exemplary patient with IDH1R132H‐mutant anaplastic astrocytoma WHO grade III as compared to three different samples of WM microglia (Figure 1C) largely confirmed the microarray data.

### Different M/M markers show a heterogeneous distribution in astrocytomas

**Figure 2:**
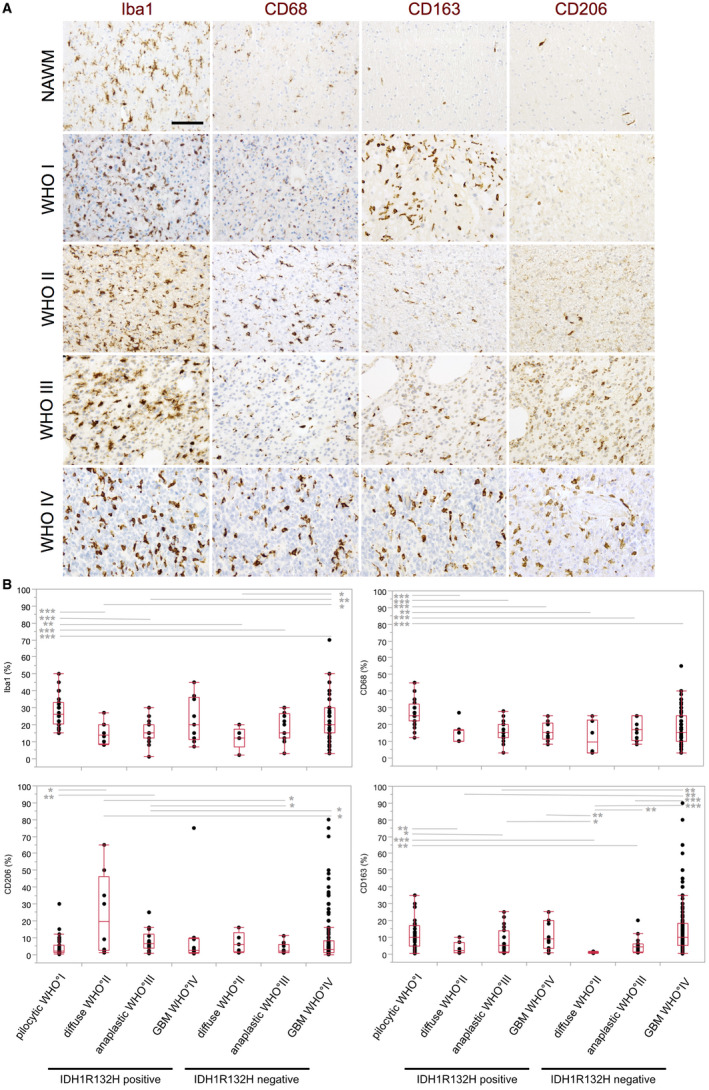
*Different M/M markers show a heterogeneous distribution in astrocytomas*. **A.** Representative immunohistochemistry stainings for Iba1, CD68, CD163 and CD206 in normal appearing white matter (NAWM) and in patients with astrocytomas of WHO grade I–IV (original magnification: 20×, scale bars = 100 µm). **B.** Iba1, CD68, CD163 and CD206 expression (see also Table 1) was statistically assessed in IDH1R132H‐mutant and ‐non‐mutant astrocytomas WHO grade I–IV using the nonparametric Wilcoxon’s test. Box and Whisker plots for positive cells (in %) are depicted. *P*‐values were indicated (*P ≤ 0.05; **P ≤ 0.01; ***P ≤ 0.001). For adjustment of the *P*‐values because of multiple testing, we used the method of Bonferroni–Holm. Only significantly different expression levels between the different entities were highlighted. Statistical analysis was performed using JMP 14.0 software (SAS).

**Figure 3:**
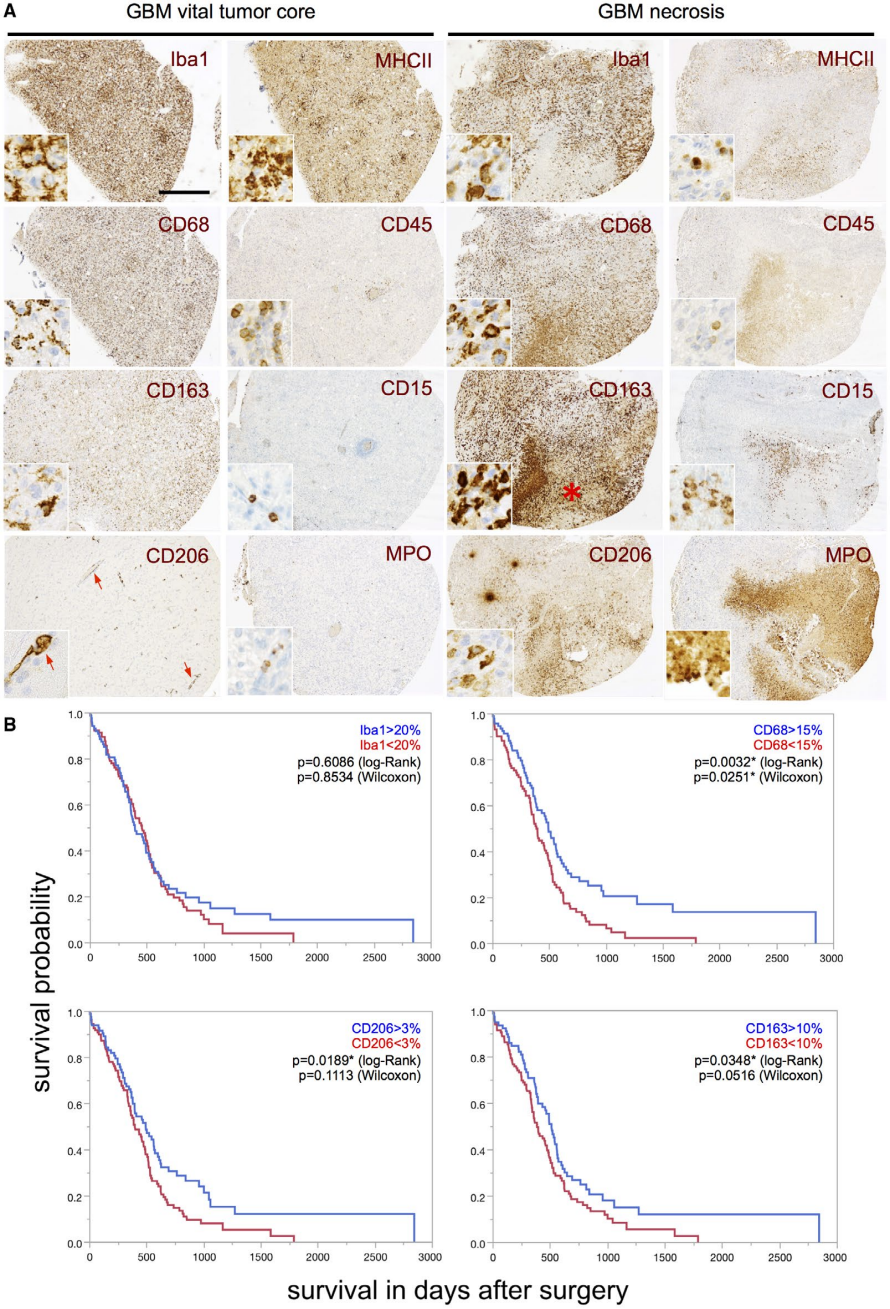
*High levels of CD68‐, CD206‐ and CD163‐positive GAMs in IDH1R132H‐non‐mutant GBM patients are associated with better survival*. **A.** Representative immunohistochemistry stainings for Iba1, CD68, CD163 and CD206 as well as MHCII, CD45, CD15 and MPO in either the vital tumor core or the perinecrotic tumor area (necrosis = red asterisk) of selected GBM patients (original magnification: 4×, scale bars = 100 µm; inserts = 40×). Red arrows indicate a predominant perivascular localization of CD206‐positive GAMs. **B.** Kaplan–Meier survival curves of IDH1R132H‐non‐mutant GBM patients were obtained performing median split for the different subpopulations of Iba1‐, CD68‐, CD163‐ and CD206‐positive GAMs (respected median splits in % depicted). Curves were compared by log–rank and Wilcoxon’s test (*P*‐values depicted).

To characterize the distribution pattern of GAMs in human IDH1R132H‐mutant and ‐non‐mutant astrocytomas WHO grade I–IV, we investigated the expression levels of the following M/M markers: Iba1 (pan‐M/M marker), CD68 (lysosomal/endosomal‐associated membrane glycoprotein; highly expressed by M/M), CD163 (scavenger receptor, proposed M2‐like marker), CD206 (macrophage mannose receptor, proposed M2‐like marker) (Figure 2A). Exemplarily, we further assessed the distribution of MHCII, CD45 and the myelomonocytic markers myeloperoxidase (MPO) and CD15 (Figure 3A).

From a technical point of view, we achieved a highly significant intra‐ (Spearmans *ρ* = 0.8729, *P* < 0.0001***) or interobserver (Spearmans *ρ* = 0.8577, *P* < 0.0001***) agreement of manual GAM quantification. Correlation analysis of repeated TMA cores of the same patients revealed a high similarity without major intraindividual differences regarding the levels of Iba1 positive cells (Figure S2A). Moreover, automated image analysis for Iba1 staining to control accuracy and precision of manual IHC quantification showed a high correlation (Figure S2B).

Statistical analysis of Iba1‐, CD68‐, CD163‐ and CD206‐positive GAMs in pilocytic astrocytomas and IDH1R132H‐mutant and ‐non‐mutant diffuse astrocytomas of WHO grade II–IV revealed highest numbers for the Iba1‐positive GAM fraction in all astrocytomas followed by the presumed pan‐M/M marker CD68 and CD163 being expressed by a slightly lower proportion of GAMs (Figure 2B, Figure S3A–D, Table 1). In summary, GAMs of all analyzed subpopulations did mostly not differ significantly across the WHO grades within the respective molecular subclasses of neither IDH1R132H‐mutant nor ‐non‐mutant diffuse astrocytomas of WHO grade II–IV. Relatively high levels of Iba1‐ and CD163‐positive GAMs were observed in IDH1R132H‐non‐mutant GBMs without showing a significant difference between the distinct molecular GBM subclasses (Figure 2B, Figure S3A–D). Also, when directly comparing lower grade astrocytomas of WHO grade II and III with GBMs, there were merely nonsignificant trends of higher GAM levels in GBMs. However, significantly more CD163‐positive GAMs were observed in IDH1R132H‐non‐mutant GBMs as compared to IDH1R132H‐non‐mutant astrocytomas of WHO grade II/III (Figure S3A–D). GAM levels of all investigated subtypes were quite heterogeneous especially in GBM patients (Figure 2B, Table 1). Comparing diffuse astrocytomas of WHO grade II–IV with pilocytic astrocytomas, both pan‐M/M markers Iba1 and CD68 as well as the presumed M2‐marker C163 were found in significantly higher levels in pilocytic astrocytomas WHO grade I (Figure 2B, Table 1). The presumed M2 markers CD163 and CD206 displayed a distinct distribution pattern in the GBM microenvironment. We detected CD163‐positive GAMs mainly in perinecrotic GBM areas, but also in the vital tumor core. CD206‐positive GAMs were generally present in low numbers in astrocytomas of all WHO grades I–IV (Figure 2, Table 1), predominantly found in perivascular localization and in perinecrotic areas (Figure 3A). Additional quantification of Iba‐1‐positive GAMs in patients with a DNA‐methylation profile of IDH‐wild‐type GBM but lower histopathological grading of WHO grade II or III (n = 3) only revealed a statistically nonsignificant trend toward lower GAM levels in the patient group with lower histopathological grading as compared to patients with IDH‐wild‐type GBM showing typical GBM features in both histopathological and DNA‐methylation‐based assessment (n = 8) (Figure S3E).

**Table 1:** GAM levels in astrocytomas of WHO grade I–IV.

	N	Iba1 (%)			C68 (%)				
		Min	Max	Median	Min	Max		Median	
pilocytic WHO°I	44	15	50	26	12	45		25	
IDH1R132H+ diffuse WHO°II	8	8	27	14	10	27		10	
IDH1R132H+ anaplastic WHO°III	19	1	30	15	3	28		15	
IDH1R132H+ GBM WHO°IV	10	7	45	20	8	25		15	
IDH1R132H‐ diffuse WHO°II	6	2	20	12	3	25		9.5	
IDH1R132H‐ anaplastic WHO°III	16	3	30	15	8	25		17	
IDH1R132H‐ GBM WHO°IV	241	3	70	20	3	55		15	
		CD206 (%)			CD163 (%)				
	N	Min	Max	Median	Min		Max		Median
pilocytic WHO°I	44	0.1	30	2	0.2		35		10
IDH1R132H+ diffuse WHO°II	8	1	65	19.5	0.5		10		2
IDH1R132H+ anaplastic WHO°III	19	0.6	25	6.5	0.5		25		5
IDH1R132H+ GBM WHO°IV	10	0.5	75	2.5	0.5		25		9
IDH1R132H‐ diffuse WHO°II	6	1	16	6	0.4		1.5		1
IDH1R132H‐ anaplastic WHO°III	16	1	11	2	0.7		20		4
IDH1R132H‐ GBM WHO°IV	241	0.02	80	3	0.1		90		10

### Association of Iba1‐, CD68‐, CD206‐ and CD163‐positive cells with glioblastoma patient survival

**Figure 4:**
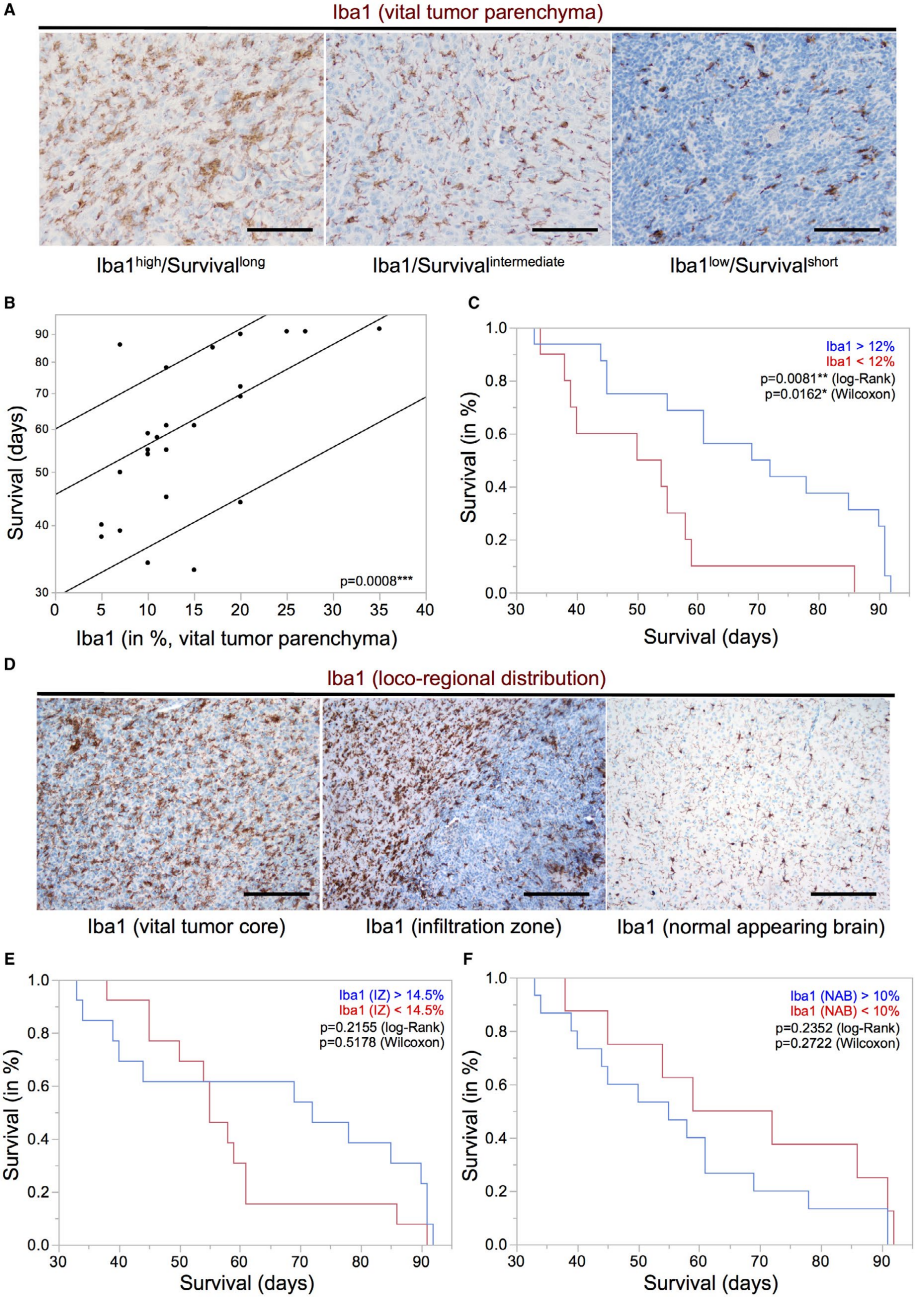
*High levels of Iba1‐positive GAMs in the vital tumor core of GBM PDOX models are associated with better survival*. **A.** Representative immunohistochemistry stainings for Iba1 in GBM patient‐derived orthotopic xenografts (PDOX) showing distinct GAM infiltration levels (original magnification: 20×, scale bars = 100 µm). **B.** Parametric survival analysis of tumor‐bearing mice (n = 26, corresponding to 12 PDOX models, 1–3 mice per model) shows a significant association of higher levels of Iba1‐positive GAMs with longer survival (*P* = 0.0008). **C.** Kaplan–Meier survival curves were obtained performing median split for Iba1 levels (high expression >12% Iba1‐positive GAM, low expression ≤12% Iba1‐positive GAM). Curves were compared by log–rank and Wilcoxon’s test (*P*‐values depicted). **D.** PDOXs displayed heterogeneous GAM distribution patterns (original magnification: 10×, scale bars = 50 µm). Kaplan–Meier survival curves of Iba1‐positive GAMs in **E.** infiltration zone (IZ) and in **F.** normal appearing brain tissue (NAB) of PDOXs were obtained performing median split [in (E/F): high expression >14.5%/10% Iba1‐positive GAMs; low expression ≤14.5%/10% Iba1‐positive GAMs] levels. Curves were compared by log–rank and Wilcoxon’s test (*P*‐values depicted).

Our IHC analyses confirmed a heterogeneous distribution pattern of GAM subpopulations in distinct tumor areas (Figure 3A). Despite the locoregional heterogeneity, we found a significant positive association of the amounts of CD68‐, CD163‐ and CD206‐positive GAM subpopulations in the vital tumor core with prolonged overall survival of IDH1R132H‐non‐mutant GBM patients performing a median split for each M/M marker (Figure 3B). As for Iba‐1 positive GAMs, this positive prognostic impact could be corroborated when strictly discriminating Iba1^high^‐ from Iba1^low^‐ tumors (best split 30%) in patients with IDH1R132H‐non‐mutant GBMs (Figure S4A) while Iba1‐median split (20%) showed a similar trend toward a positive correlation with patient survival yet missing statistical significance. GBM PDOX models likewise showed a correlation of beneficial survival with high amounts of Iba1‐positive cells in the vital tumor core (Figure 4A to C). In PDOX models, GAM levels were not related to an invasive or noninvasive growth pattern *(data not shown).* Interestingly, high levels of GAMs in the GBM IZ or NAB did not correlate with overall survival neither in PDOX models (Figure 4D–F) nor in the large cohort of IDH1R132H‐non‐mutant GBM patients (assessed by Iba1‐, CD68‐, CD163‐, CD206‐IHC, Figure S4B–E).

### The transcriptome profile of GAMs is functionally related to cancer‐associated M/M cell motility and immune cell communication regulated by TNFα signaling

**Figure 5:**
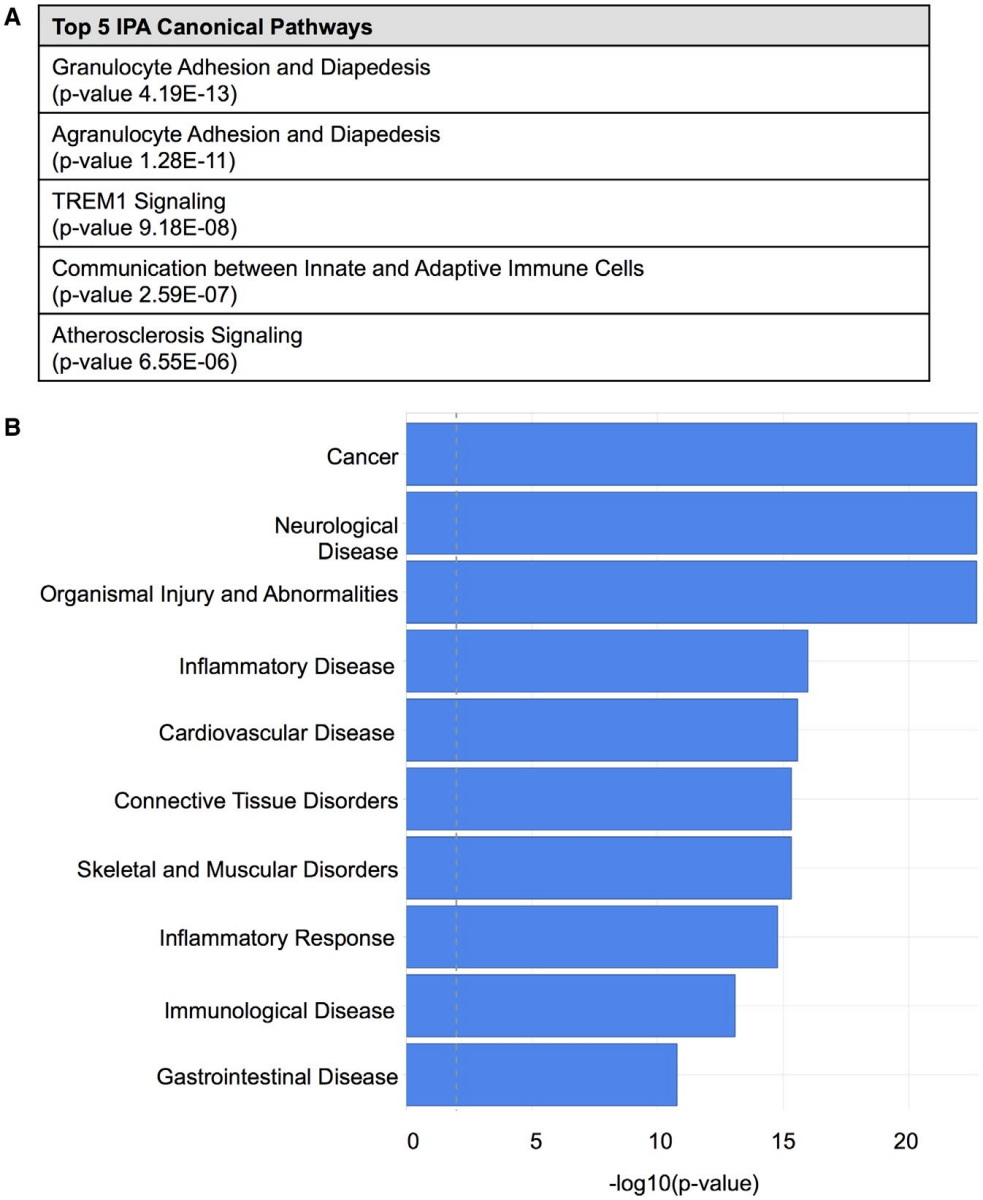
*Ingenuity pathway analysis (IPA) reveals molecular pathways and processes involved in the immunological polarization profile of GAMs*. **A.** The top five ranked IPA canonical pathways and **B** the top ten ranked diseases and disorders detected by Ingenuity in the dataset of the Affymetrix GeneChip Human gene 2.0 ST array (GAMs compared to normal WM microglia) are depicted. The threshold (dashed line) corresponds to a *P*‐value of 0.01.

Ingenuity Pathway Analysis (IPA) (complete IPA: Table S1) revealed, that genes differentially regulated in GAMs as compared to reference normal WM microglia were enriched for factors involved in canonical pathways of immunological processes (Figure 5A) and phagocytosis (Table S2). IPA identified a relation to ten top diseases including cancer, neurological disease, inflammatory disease and response and immunological disease (Figure 5B). IPA upstream regulator analysis retrieved 10 potential molecules whose differential regulation could explain the gene expression profile obtained comparing GAMs with WM microglia. Most of these presumably activated upstream regulators belong to the cytokine family with TNF showing highest significant activation levels in GAMs (Table S3). Corroborating the IPA findings, DAVID analysis revealed mostly immunologically related annotation clusters (complete DAVID analysis: Table S4).

## Discussion

There is conflicting data about the prognostic impact of the innate immune system including GAMs in gliomas 15, 23, 41, 44. An immunosuppressive state with decreased adaptive 4, 35, 42 and innate 8, 12, 17 immune response in glioma patients has been claimed since decades. Although a body of literature is trying to characterize potential pro‐ or anti‐neoplastic GAM functions, a direct link between GAM levels and patient survival was hardly assessed systematically. So far, there is no consensus about the immunological phenotype and prognostic role of different GAM subpopulations 15. Against the background of recent diagnostic improvements including molecular and DNA‐methylation profiling 7, we investigated the prognostic impact of different GAM subpopulations in a clearly defined patient cohort applying the WHO criteria of 2016, immunohistochemical analysis of IDH1R132H‐mutation status and exemplarily DNA‐methylation‐based classification of selected patients.

In astrocytomas of WHO grades I–IV, we confirmed the findings of previous studies 50 observing highest levels of GAMs in pilocytic astrocytomas WHO grade I (Figure 2), an entity that has to be considered separately from diffuse astrocytomas of WHO grades II to IV. While several publications describe increasing GAM levels depending on the WHO grade of diffuse gliomas 15, we merely detected nonsignificant trends of increasing levels of Iba1‐ and CD68‐positive GAMs (both classical pan‐M/M marker) with increasing WHO grades II to IV in the distinct molecular subclasses of IDH1R132H‐mutant and ‐non‐mutant diffuse astrocytomas. In GBMs, we detected relatively high levels of Iba1‐ and CD163‐positive GAMs without showing a relevant difference between the molecular subclasses of IDH1R132H‐mutant and non‐mutant GBMs (Figure 2B, Figure S3A–D). Within our cohort of DNA‐methylation‐based classified IDH‐wild‐type GBMs, we could likewise only detect a nonsignificant trend toward lower Iba1‐positive GAM levels in patients with — discordant to the DNA‐methylation profiling — lower histopathological grading (Figure S3E). A limitation of this additional epigenetically defined patient cohort was the relatively low number of patient cases. In line with previous studies investigating intertumoral heterogeneity of GBM patients 52, GAM levels were quite heterogeneous in the different patients investigated in our largest TMA cohort of 241 IDH1R132H‐non‐mutant GBM patients. The scavenger receptor CD163 is considered as an anti‐inflammatory molecule indicating M2‐polarization. The similarly high expression of the pan‐M/M marker Iba1 and CD163 in the very distinct entities of pilocytic astrocytomas of WHO grade I and IDH1R132H‐non‐mutant GBMs, as also shown by others 31, challenges a general M1/M2 concept of M/M polarization in gliomas.

Most interestingly, high intratumoral levels of GAMs of all M/M markers (including both presumptive M2 markers CD163 and CD206) were associated with a better overall survival in IDH1R132H‐non‐mutant GBM patients (Figure 3B, Figure S4A) independent of the aforementioned expression levels in WHO grades II–IV with a tendency toward highest GAM amounts in GBM patients as compared to lower grade diffuse astrocytomas (Figure 2B, Figure S3). Our survival data, therefore, also question the common interpretation of increased numbers of GAMs with expression of M2 markers as a surrogate for tumor progression 15. Remarkably, both putative M2 markers CD163 and CD206 showed a very distinct expression profile and locoregional distribution pattern (Figure 2 and 3A). In addition to the different GAM subpopulations investigated in our study, all showing a positive prognostic impact independent of differing expression levels and intratumoral spatial distribution (eg, predominant perivascular or perinecrotic localization), we could confirm the finding of our previous study investigating a CD74‐positive GAM subpopulation likewise demonstrating a positive association of high GAM levels with patients’ overall survival 53. The survival data could additionally be corroborated for Iba1‐positive GAMs in PDOX models (Figure 4A to C). Remarkably, our PDOX models performed in NOD/SCID mice display a per se immunocompromised microenvironment that still needs further investigation. However, GAM activation in comparable models was previously reported 45 and GAMs did show a rather activated, amoeboid morphology in comparison to microglia in NAB (Figure 4). Irrespectively of the aforementioned issue, our survival data were surprising especially when GAM subpopulations expressing the presumptive M2 markers CD163‐ and CD206 were considered as they are assumed to display anti‐inflammatory, pro‐neoplastic and phagocytic functions, the latter being a common feature in the GBM microenvironment around pathognomonic necroses (Figure 3A). Together with the transcriptome analyses of MACS®‐selected GAMs, indicating a functional relation to cancer‐associated M/M motility, immune cell communication and phagocytosis (Figure 5, Tables [Link], [Link], [Link], [Link]–S4), the presumptive M2‐like GAM subpopulations might rather be important for phagocytosis and antigen presentation in the GBM microenvironment than representing an anti‐inflammatory and potentially tumor‐promoting GAM phenotype.

**Figure 6:**
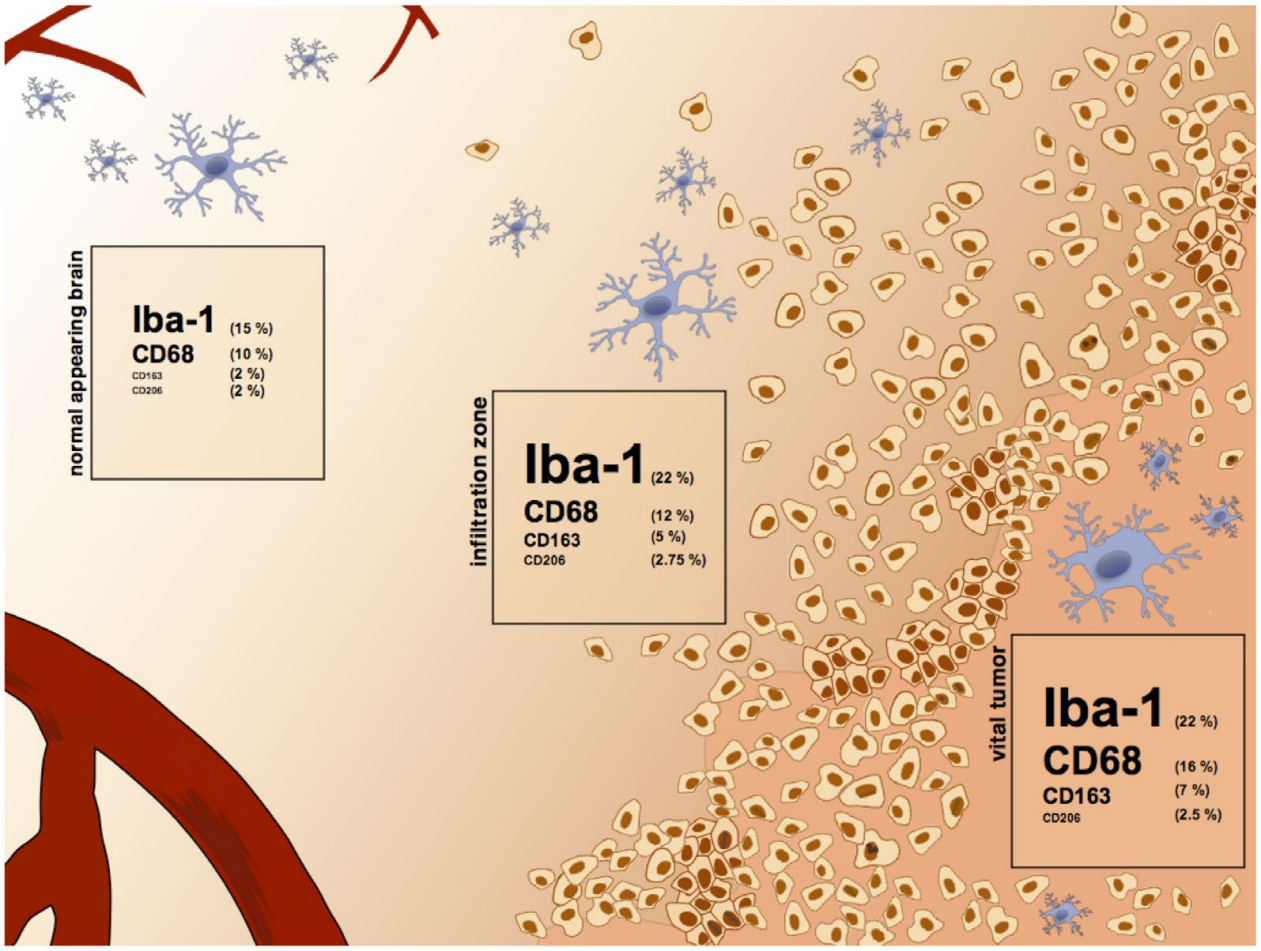
*Distribution of GAM subpopulations*. The figure illustrates the heterogeneous distribution of different GAM subtypes in the microenvironment of IDH1R132H‐non‐mutant GBMs. Iba1‐, CD68‐, CD206‐ and CD163‐positive GAMs were quantified by IHC in the vital tumor center, the infiltration zone and the normal appearing brain tissue (see Figure S5 for statistical analyses).

To decipher potential GAM functions in the GBM microenvironment, we assessed the immunological phenotype of MACS®‐selected GAMs. We identified a rather mixed polarization phenotype with parallel expression of presumptive M1 and M2 markers (Figure 1) and without evidence for an unequivocal M1 or M2 polarization state of GAMs according to the M1/2 model. CD11b‐selected GAMs displayed an immune signature with even a predominance of M1 cytokines and parallel upregulation of M2 molecules (Figure 1), most likely reflecting pathognomonic features of the microenvironment (eg, phagocytosis ‐> *MRC1, CD163, MSR1, *Table S2). Further, our bioinformatic analyses suggest the pro‐inflammatory TNFα pathway as a prominent upstream regulator pathway contributing to the identified GAM signature (Table S3). TNFα is one of the major pro‐inflammatory M1 cytokines and displays cytotoxic antitumor functions 19, although its pleiotropic effects may also act in a tumor‐promoting manner in gliomas 29. A recent study identified GAM‐derived TNFα as significant regulator of therapeutic response to oncolytic HSV‐1 therapy in GBM patients 30. However, another study in murine glioma model found a decreased TNFα secretion in GAMs, interpreted as functional impairment 22. Of note, the gene expression data provide a mean level of expression restricted to a subpopulation of GAMs selected with the pan‐M/M marker CD11b. Thus, the analysis might miss potentially divergent polarization profiles of smaller GAM subpopulations with specialized functions (and hence specific polarization patterns). As we observed a heterogeneous distribution of different GAM subpopulations (Figures 2 and 3), further analyses concerning their potentially distinct functional properties are needed. However, the concept of a mixed immune phenotype of GAMs is recently corroborated by others 12, 20, 43, 47, 49 likewise failing to identify a clear M1 or M2 phenotype. Also, some studies are lacking the awareness for the detection of pro‐inflammatory M1 cytokines in GAMs while almost exclusively emphasizing on parallel overexpressed M2 cytokines 52, the latter considered to comprise tumor‐promoting functions as known from TAMs in non‐CNS cancer entities. Altogether, this suggests that the M1/M2 model might be an over‐simplification in CNS immunobiology 43. The classification of the prognostic impact of GAMs based on a limited panel of predefined cytokines attributing a so‐called M1 or M2 polarization state in GAMs as performed even recently 52, 54 seems therefore highly questionable, especially without considering different GAM subpopulations, regional heterogeneities and clinical patient data. As a summary, Figure 6 and Figure S5 illustrate the potential intratumoral distribution of different GAM subtypes in the GBM microenvironment according to our immunohistochemical observations.

Another important finding regarding the survival analysis was the relevance of spatial information discriminating GAMs in the vital tumor center from those in the IZ. GAMs in the vital tumor showed a significant positive prognostic impact in both GBM patients and PDOX models in contrast to GAMs evaluated separately in the IZ of our human GBM patient cohort (Figure S4B–E) and PDOX models (Figure 4E and F). These findings point toward a distinct role of intratumoral GAMs that have so far not been assessed separately from GAMs in the IZ in most studies. In fact, a recent publication showed a distinct treatment response to inhibition of colony stimulating factor 1 receptor (CSF‐1R) of peritumoral GAMs in GBM mouse model 40. Thus, the critical association of the spatial distribution of GAMs with the prognostic impact might explain seemingly conflicting findings as compared to previous studies and database analyses suggesting a rather unfavorable prognostic impact of GAMs. Although there are attempts to correct datasets for contamination bias *in silico* 52, missing information about the exact expression levels and spatial distribution of GAM at the level of detail as obtained in our study (eg, vital tumor tissue analyzed separately from IZ) is a potential bias that might explain conflicting data, especially when data collection is based on homogenized tumor tissue.

In conclusion, this study provides evidence that the immune polarization phenotype of GAMs might be distinct from TAMs in non‐CNS cancers. Intratumoral GAMs display both M1 and M2 genes with even a predominance of presumed M1 cytokines. The additional upregulation of presumed M2 molecules may reflect pathognomonic features of the GBM microenvironment. Despite their heterogeneous distribution, different GAM subtypes are altogether associated with a favorable overall survival in patients with IDH1R132H‐non‐mutant GBMs. This could also be confirmed in PDOX models. The localization of GAMs inside the vital tumor core seemed to be critical for the statistical evaluation of survival analysis. Taken together, our findings present GAMs as an interesting tool for prognostic evaluation and shed a new light on therapeutic studies that are focusing on a potential GAM depolarization toward a presumably more favorable phenotype as investigated in preclinical 40 and clinical trials (latest approaches reviewed in 41, 44).

## Funding

PSZ obtained grants from Patenschaftsmodell Universität Frankfurt (an intramural funding that was attributed for this project for PSZ, grant number F11/14_R114/2014) during the conduct of the study. MM would like to thank the Luxembourg National Research Fund (FNR) for the support (FNR PEARL P16/BM/11192868 grant). ILUMINATE (FKZ:031 B0006C) funded by German Ministry for Education and Research (BMBF), Project Management Juelich (PTJ), supported AG and FF.

## Conflicts of interest

No conflict of interest regarding the publication of this article.

## Author contributions

Conception and design of the work: PSZ, PNH and MM. Acquisition, analysis and interpretation of data: PSZ, CP, AG, JZ, AI, AM, TK, KF, MME, SB, AEB, PB, EI, AG, MLH, MAV, KF, FF, JPS, JW, WS, SPN, PNH, MM. Drafting of the manuscript: PSZ, PNH and MM. Critical revision for important intellectual content: PSZ, CP, AG, JZ, AI, AM, TK, KF, MME, SB, AEB, PB, EI, AG, MLH, MAV, KF, FF, JPS, JW, WS, SPN, PNH, MM All authors approved the final version of the manuscript and agreed to all aspects of the work in ensuring that questions related to the accuracy or integrity of any part of the work are appropriately investigated and resolved.

## Supporting information


**Figure S1.** *CD11b‐*MACS®* selected GAMs show a high purity*. **A.** GAMs and primary tumor cell cultures (PC) were extracted from fresh human glioma tissue via CD11b‐based MACS® or enzymatic and mechanical dissociation, respectively. **B.** In individual patients with high‐grade astrocytomas (GBM1‐3, astrocytoma WHO grade III), corresponding protein lysates of GAMs, PCs and the whole glioma tissue (T) were investigated for Iba1‐expression. Actin served as positive control. The CD11b‐MACS® selected GAM suspension **C** was analyzed by immunocytochemistry stainings for the classical M/M markers **D.** Iba1, **E.** MHCII, **F**. CD45 as well as (G) GFAP as a negative control and indicator for a potential contamination with tumor cells (original magnification b–h: 20×, scale bar = 100 µm).

**Figure S2.** *Manual and automated quantification of Iba1‐immunohistochemistr*y. **A**. Correlation analysis revealing a high similarity of Iba1 levels between repeated tissue cores of individual patients of our TMA patients’ cohort (Spearmans *ρ* = 0.7121, *P* < 0.0001). **B**. Correlation analysis revealing that manual and automated quantification of Iba1‐immunohistochemistry strongly positively correlate in our TMA patients’ cohort (r2 = 0.672; *P* < 0.0001).

**Figure S3.** *Expression of Iba1‐positive GAMs in patients with diffuse lower grade astrocytomas compared to GBMs*. **A–D**. Iba1, CD68, CD163 and CD206 levels were statistically assessed in IDH1R132H‐mutant and ‐non‐mutant lower grade astrocytomas (WHO II/III) compared to GBMs using the nonparametric Bonferroni's multiple comparisons test. Only significantly different expression levels between the different entities were highlighted. Statistical analysis was performed using GraphPad Prism 7 software. **E**. Iba1‐positive GAMs were quantified in methylation‐based classified IDH‐wild‐type GBMs with discordantly lower histopathological grading (n = 3) in comparison to patients with concordant classification of IDH‐wild‐type in both histopathology and methylation profiling (n = 8). Aligned dot plots for Iba1‐positive GAMs (in %) are depicted. The relative amount of positive cells was statistically assessed using the nonparametric Mann–Whitney test (*P* = 0.1333).

**Figure S4.** *High levels of Iba1‐positive GAMs in IDH1R132H‐non‐mutant GBM patients are associated with better survival*. **A.** Kaplan–Meier survival curves of IDH1H132R‐non‐mutant GBM patients were obtained by performing best split (high expression >30% Iba1‐positive GAMs; low expression ≤30% Iba1‐positive GAMs) additionally to median split (20%, depicted in Figure 3B). **B–D.** Kaplan–Meier survival curves of Iba1‐, CD68‐, CD206‐ and CD163 positive GAMs in the infiltration zone (IZ) of GBM patients were obtained by performing median splits as indicated in the figure. Curves were compared by log–rank and Wilcoxon’s test (*P*‐values depicted).

**Figure S5.** *Distribution of GAM subpopulations*. This figure depicts the statistical analyses performed for the illustration of Figure 6. **A.** Iba1‐, **B.** CD68‐, **C.** CD206‐ and **D.** CD163‐positive GAMs were quantified by IHC in the vital tumor center (T), the infiltration zone (IZ) and the normal appearing brain tissue (NAB) of IDH1R132H‐non‐mutant GBMs. Box and Whisker plots for positive cells (in %) are depicted. *P*‐values were indicated (*P ≤ 0.05; **P ≤ 0.01; ***P ≤ 0.001) after performing nonparametric Dunn testing. Only significantly different expression levels between the localizations were depicted. Statistical analysis was performed using JMP 14.0 software (SAS).

**Table S1.** *Ingenuity pathway analysis reveals different functions of GAMs. *Complete ingenuity pathway analysis (IPA) analysis for regulated functions of the microarray dataset, comparing GAMs to normal WM microglia.

**Table S2.** *Ingenuity pathway analysis indicates a phagocytic and migratory GAM phenotype*. IPA analysis for the Diseases or Functions Annotation of phagocytic engulfment of the microarray dataset, comparing GAMs to normal WM microglia.

**Table S3.** *Ingenuity pathway analysis reveals upstream regulators of GAM polarization*. Ingenuity pathway analyses reveals top 10 upstream regulators leading to the particular gene expression phenotype in GAMs in comparison to normal WM microglia. An upstream regulator can deemed significant if associated *P*‐value of overlap is less than 1^E^‐02. Furthermore, it is considered as activated if associated *z*‐score is greater than 2 (or alternatively inhibited is *z*‐score is less than −2).

**Table S4.** *Complete DAVID analysis reveals molecular pathways involved in the immunological polarization profile of GAMs*. Complete DAVID analysis for GO enrichment of genes of the microarray dataset, comparing GAMs to normal WM microglia.

## References

[1] Baumgarten P , Blank AE , Franz K , Hattingen E , Dunst M , Zeiner P *et al* (2016) Differential expression of vascular endothelial growth factor A, its receptors VEGFR‐1, ‐2, and ‐3 and co‐receptors neuropilin‐1 and ‐2 does not predict bevacizumab response in human astrocytomas. Neuro Oncol 8:173–183. 10.1093/neuonc/nov288 PMC4724185 26627848

[2] Berghoff AS , Kiesel B , Widhalm G , Rajky O , Ricken G , Wöhrer A *et al* (2015) Programmed death ligand 1 expression and tumor‐infiltrating lymphocytes in glioblastoma. Neuro Oncol 17:1064–1075. 25355681 10.1093/neuonc/nou307 PMC4490866

[3] Bougnaud S , Golebiewska A , Oudin A , Keunen O , Harter PN , Mäder L *et al* (2016) Molecular crosstalk between tumour and brain parenchyma instructs histopathological features in glioblastoma. Oncotarget 7:31955–31971. 27049916 10.18632/oncotarget.7454 PMC5077988

[4] Brooks WH , Netsky MG , Normansell DE , Horwitz DA (1972) Depressed cell‐mediated immunity in patients with primary intracranial tumors. Characterization of a humoral immunosuppressive factor. J Exp Med 136:1631–1647. 4345108 10.1084/jem.136.6.1631 PMC2139329

[5] Budhu A , Forgues M , Ye QH , Jia HL , He P , Zanetti KA *et al* (2006) Prediction of venous metastases, recurrence, and prognosis in hepatocellular carcinoma based on a unique immune response signature of the liver microenvironment. Cancer Cell 10:99–111. 16904609 10.1016/j.ccr.2006.06.016

[6] Burnett GT , Weathersby DC , Taylor TE , Bremner TA (2008) Regulation of inflammation‐ and angiogenesis‐related gene expression in breast cancer cells and co‐cultured macrophages. Anticancer Res 28:2093–2099. 18751381

[7] Capper D , Jones DTW , Sill M , Hovestadt V , Schrimpf D , Sturm D (2018) DNA methylation‐based classification of central nervous system tumours. Nature 555:469–474. 29539639 10.1038/nature26000 PMC6093218

[8] Dubinski D , Wölfer J , Hasselblatt M , Schneider‐Hohendorf T , Bogdahn U , Stummer W *et al* (2016) CD4+ T effector memory cell dysfunction is associated with the accumulation of granulocytic myeloid‐derived suppressor cells in glioblastoma patients. Neuro Oncol 18:807–818. 26578623 10.1093/neuonc/nov280 PMC4864257

[9] Durafourt BA , Moore CS , Zammit DA , Johnson TA , Zaguia F , Guiot MC *et al* (2012) Comparison of polarization properties of human adult microglia and blood‐derived macrophages. Glia 60:717–727. 22290798 10.1002/glia.22298

[10] Forssell J , Oberg A , Henriksson ML , Stenling R , Jung A , Palmqvist R (2007) High macrophage infiltration along the tumor front correlates with improved survival in colon cancer. Clin Cancer Res 13:1472–1479. 17332291 10.1158/1078-0432.CCR-06-2073

[11] Fridman WH , Pagès F , Sautès‐Fridman C , Galon J (2012) The immune contexture in human tumours: impact on clinical outcome. Nat Rev Cancer 12:298–306. 22419253 10.1038/nrc3245

[12] Gabrusiewicz K , Rodriguez B , Wei J , Hashimoto Y , Healy LM , Maiti SN *et al* (2016) Glioblastoma‐infiltrated innate immune cells resemble M0 macrophage phenotype. JCI Insight 1:pii: e85841. 10.1172/jci.insight.85841 PMC4784261 26973881

[13] Galluzzi L , Vacchelli E , Bravo‐San Pedro JM , Buqué A , Senovilla L , Baracco EE *et al* (2014) Classification of current anticancer immunotherapies. Oncotarget 5:12472–12508. 25537519 10.18632/oncotarget.2998 PMC4350348

[14] Gentles AJ , Newman AM , Liu CL , Bratman SV , Feng W , Kim D *et al* (2015) The prognostic landscape of genes and infiltrating immune cells across human cancers. Nat Med 21:938–945. 26193342 10.1038/nm.3909 PMC4852857

[15] Gieryng A , Pszczolkowska D , Walentynowicz KA , Rajan WD , Kaminska B (2017) Immune microenvironment of gliomas. Lab Invest 97:498–518. 10.1038/labinvest.2017.19. 28287634

[16] Guiducci C , Vicari AP , Sangaletti S , Trinchieri G , Colombo MP (2005) Redirecting *in vivo* elicited tumor infiltrating macrophages and dendritic cells towards tumor rejection. Cancer Res 65:3437–3446. 15833879 10.1158/0008-5472.CAN-04-4262

[17] Han S , Zhang C , Li Q , Dong J , Liu Y , Huang Y *et al* (2014) Tumour‐infiltrating CD4(+) and CD8(+) lymphocytes as predictors of clinical outcome in glioma. Br J Cancer 110:2560–2568. 24691423 10.1038/bjc.2014.162 PMC4021514

[18] Hanahan D , Weinberg RA (2011) Hallmarks of cancer: the next generation. Cell 144:646–674. 21376230 10.1016/j.cell.2011.02.013

[19] Hao C , Parney IF , Roa WH , Turner J , Petruk KC , Ramsay DA (2002) Cytokine and cytokine receptor mRNA expression in human glioblastomas: evidence of Th1, Th2 and Th3 cytokine dysregulation. Acta Neuropathol 103:171–178. 11810184 10.1007/s004010100448

[20] Hattermann K , Sebens S , Helm O , Schmitt AD , Mentlein R , Mehdorn HM *et al* (2014) Chemokine expression profile of freshly isolated human glioblastoma‐associated macrophages/microglia. Oncol Rep 32:270–276. 24859792 10.3892/or.2014.3214

[21] Heimberger AB , Abou‐Ghazal M , Reina‐Ortiz C , Yang DS , Sun W , Qiao W *et al* (2008) Incidence and prognostic impact of FoxP3+ regulatory T cells in human gliomas. Clin Cancer Res 14:5166–5172. 18698034 10.1158/1078-0432.CCR-08-0320

[22] Kennedy BC , Maier LM , D’Amico R , Mandigo CE , Fontana EJ , Waziri A *et al* (2009) Dynamics of central and peripheral immunomodulation in a murine glioma model. BMC Immunol 18;10:11. 10.1186/1471-2172-10-11 PMC2654428 19226468

[23] Kennedy BC , Showers CR , Anderson DE , Anderson L , Canoll P , Bruce JN *et al* (2013) Tumor‐associated macrophages in glioma: friend or foe? J Oncol 2013:486912. 10.1096/fj.08-109611. PMC3664503 23737783

[24] Kim YH , Jung TY , Jung S , Jang WY , Moon KS , Kim IY *et al* (2012) Tumour‐infiltrating T‐cell subpopulations in glioblastomas. Br J Neurosurg 26:21–27. 21707245 10.3109/02688697.2011.584986

[25] Kortylewski M , Kujawski M , Wang T , Wei S , Zhang S , Pilon‐Thomas S *et al* (2005) Inhibiting Stat3 signaling in the hematopoietic system elicits multicomponent antitumor immunity. Nat Med 11:1314–1321. 16288283 10.1038/nm1325

[26] Lohr J , Ratliff T , Huppertz A , Ge Y , Dictus C , Ahmadi R *et al* (2011) Effector T‐cell infiltration positively impacts survival of glioblastoma patients and is impaired by tumor‐derived TGF‐β. Clin Cancer Res 17:4296–4308. 21478334 10.1158/1078-0432.CCR-10-2557

[27] Luo Y , Zhou H , Krueger J , Kaplan C , Lee SH , Dolman C *et al* (2006) Targeting tumor‐associated macrophages as a novel strategy against breast cancer. J Clin Invest 116:2132–2141. 16862213 10.1172/JCI27648 PMC1513049

[28] Mantovani A , Sica A , Sozzani S , Allavena P , Vecchi A , Locati M (2004) The chemokine system in diverse forms of macrophage activation and polarization. Trends Immunol 25:677–686. 15530839 10.1016/j.it.2004.09.015

[29] McFarland BC , Hong SW , Rajbhandari R , Twitty GB Jr , Gray GK , Yu H *et al* (2013) NF‐κB‐induced IL‐6 ensures STAT3 activation and tumor aggressiveness in glioblastoma. PLoS One 8:e78728. 24244348 10.1371/journal.pone.0078728 PMC3823708

[30] Meisen WH , Wohleb ES , Jaime‐Ramirez AC , Bolyard C , Yoo JY , Russell L *et al* (2015) The impact of macrophage‐ and microglia‐secreted TNFα on oncolytic HSV‐1 therapy in the glioblastoma tumor microenvironment. Clin Cancer Res. 15;21:3274–3285. 10.1158/1078-0432.CCR-14-3118 PMC4780415 25829396

[31] Mignogna C , Signorelli F , Vismara MF , Zeppa P , Camastra C , Barni T *et al* (2016) A reappraisal of macrophage polarization in glioblastoma: histopathological and immunohistochemical findings and review of the literature. Pathol Res Pract 212:491–499. 27101800 10.1016/j.prp.2016.02.020

[32] Mills CD , Kincaid K , Alt JM , Heilman MJ , Hill AM (2000) M‐1/M‐2 macrophages and the Th1/Th2 paradigm. J Immunol 164:6166–6173.10843666

[33] Mittelbronn M , Simon P , Löffler C , Capper D , Bunz B , Harter P *et al* (2007) Elevated HLA‐E levels in human glioblastomas but not in grade I to III astrocytomas correlate with infiltrating CD8+ cells. J Neuroimmunol 189:50–58. 17675252 10.1016/j.jneuroim.2007.07.002

[34] Mittelbronn M , Meyermann R (2010) CNS – immune system interplay unter normal and pathological conditions. In: Immune biology of brain tumours, Stavrou DK , Hagel C (eds), Dustri: Oberhaching‐Munich.

[35] Morford LA , Elliott LH , Carlson SL , Brooks WH , Roszman TL (1979) T cell receptor‐mediated signaling is defective in T cells obtained from patients with primary intracranial tumors. J Immunol 159:4415–4425. 9379040

[36] Niclou SP , Danzeisen C , Eikesdal HP , Wiig H , Brons NH , Poli AM *et al* (2008) A novel eGFP‐expressing immunodeficient mouse model to study tumor‐host interactions. FASEB J 22:3120–3128. 10.1096/fj.08-109611. 18495755 PMC2518261

[37] Ohno S , Inagawa H , Dhar DK , Fujii T , Ueda S , Tachibana M (2003) The degree of macrophage infiltration into the cancer cell nest is a significant predictor of survival in gastric cancer patients. Anticancer Res 23:5015–5022. 14981961

[38] Platten M , Kretz A , Naumann U , Aulwurm S , Egashira K , Isenmann S * et al* (2003) Monocyte chemoattractant protein‐1 increases microglial infiltration and aggressiveness of gliomas. Ann Neurol 54:388–392. 12953273 10.1002/ana.10679

[39] Pollard JW (2004) Tumour‐educated macrophages promote tumour progression and metastasis. Nat Rev Cancer 4:71–78. 10.1038/nrc1256. 14708027

[40] Pyonteck SM , Akkari L , Schuhmacher AJ , Bowman RL , Sevenich L , Quail DF * et al* (2013) CSF‐1R inhibition alters macrophage polarization and blocks glioma progression. Nat Med 19:1264–1272. 24056773 10.1038/nm.3337 PMC3840724

[41] Roesch S , Rapp C , Dettling S , Herold‐Mende C (2018) When immune cells turn bad‐tumor‐associated microglia/macrophages in glioma. Int J Mol Sci 19(2):E436. 10.3390/ijms19020436. 29389898 PMC5855658

[42] Roszman TL , Brooks WH (1980) Immunobiology of primary intracranial tumours. III. Demonstration of a qualitative lymphocyte abnormality in patients with primary brain tumours. Clin Exp Immunol 39:395–402. 6966992 PMC1538062

[43] Selenica ML , Alvarez JA , Nash KR , Lee DC , Cao C , Lin X * et al* (2013) Diverse activation of microglia by chemokine (C‐C motif) ligand 2 overexpression in brain. J Neuroinflammation 10:86. 10.1186/1742-2094-10-86. 23866683 PMC3726363

[44] Sevenich L (2018) Brain‐resident microglia and blood‐borne macrophages orchestrate central nervous system inflammation in neurodegenerative disorders and brain cancer. Front Immunol 9:697. 10.3389/fimmu.2018.00697. 29681904 PMC5897444

[45] Shi Y , Zhou Ping YF , W, He ZC Chen C, Bian BS, *et al* (2017) Tumour‐associated macrophages secrete pleiotrophin to promote PTPRZ1 signalling in glioblastoma stem cells for tumour growth. Nat Commun 8:15080. 10.1038/ncomms15080. 28569747 PMC5461490

[46] Solinas G , Schiarea S , Liguori M , Fabbri M , Pesce S , Zammataro L * et al* (2010) Tumor‐conditioned macrophages secrete migration‐stimulating factor: a new marker for M2‐polarization, influencing tumor cell motility. J Immunol 185:642–652. 20530259 10.4049/jimmunol.1000413

[47] Stables MJ , Shah S , Camon EB , Lovering RC , Newson J , Bystrom J * et al* (2011) Transcriptomic analyses of murine resolution‐phase macrophages. Blood 118:e192–e208. 22012065 10.1182/blood-2011-04-345330 PMC5362087

[48] Stupp R , Hegi ME , Gorlia T , Erridge SC , Perry J , Hong YK * et al* (2014) Cilengitide combined with standard treatment for patients with newly diagnosed glioblastoma with methylated MGMT promoter (CENTRIC EORTC 26071–22072 study): a multicentre, randomised, open‐label, phase 3 trial. Lancet Oncol 15:1100–1108. 25163906 10.1016/S1470-2045(14)70379-1

[49] Szulzewsky F , Pelz A , Feng X , Synowitz M , Markovic D , Langmann T * et al* (2015) Glioma‐associated microglia/macrophages display an expression profile different from M1 and M2 polarization and highly express Gpnmb and Spp1. PLoS One 10:e0116644. 25658639 10.1371/journal.pone.0116644 PMC4320099

[50] Tanaka Y , Sasaki A , Ishiuchi S , Nakazato Y (2008) Diversity of glial cell components in pilocytic astrocytoma. Neuropathology 28:399–407. 18312545 10.1111/j.1440-1789.2008.00896.x

[51] Tsutsui S , Yasuda K , Suzuki K , Tahara K , Higashi H , Era S (2005) Macrophage infiltration and its prognostic implications in breast cancer: the relationship with VEGF expression and microvessel density. Oncol Rep 14:425–431. 16012726

[52] Wang Q , Hu B , Hu X , Kim H , Squatrito M , Scarpace L * et al* (2017) Tumor evolution of glioma‐intrinsic gene expression subtypes associates with immunological changes in the microenvironment. Cancer Cell 32:42–56.e6. 28697342 10.1016/j.ccell.2017.06.003 PMC5599156

[53] Zeiner PS , Preusse C , Blank AE , Zachskorn C , Baumgarten P , Caspary L * et al* (2015) MIF receptor CD74 is restricted to microglia/macrophages, associated with a M1‐polarized immune milieu and prolonged patient survival in gliomas. Brain Pathol 25:491–504. 25175718 10.1111/bpa.12194 PMC8029437

[54] Zhou W , Ke SQ , Huang Z , Flavahan W , Fang X , Paul J * et al* (2015) Periostin secreted by glioblastoma stem cells recruits M2 tumour‐associated macrophages and promotes malignant growth. Nat Cell Biol 17:170–182. 25580734 10.1038/ncb3090 PMC4312504

