# Supplementary figures and images for: Distribution and prognostic impact of microglia/macrophage subpopulations in gliomas
Source: Brain Pathol. 2019 Jan 15;29(4):513–29. doi: 10.1111/bpa.12690 (PMC6849857; doi:10.1111/bpa.12690)

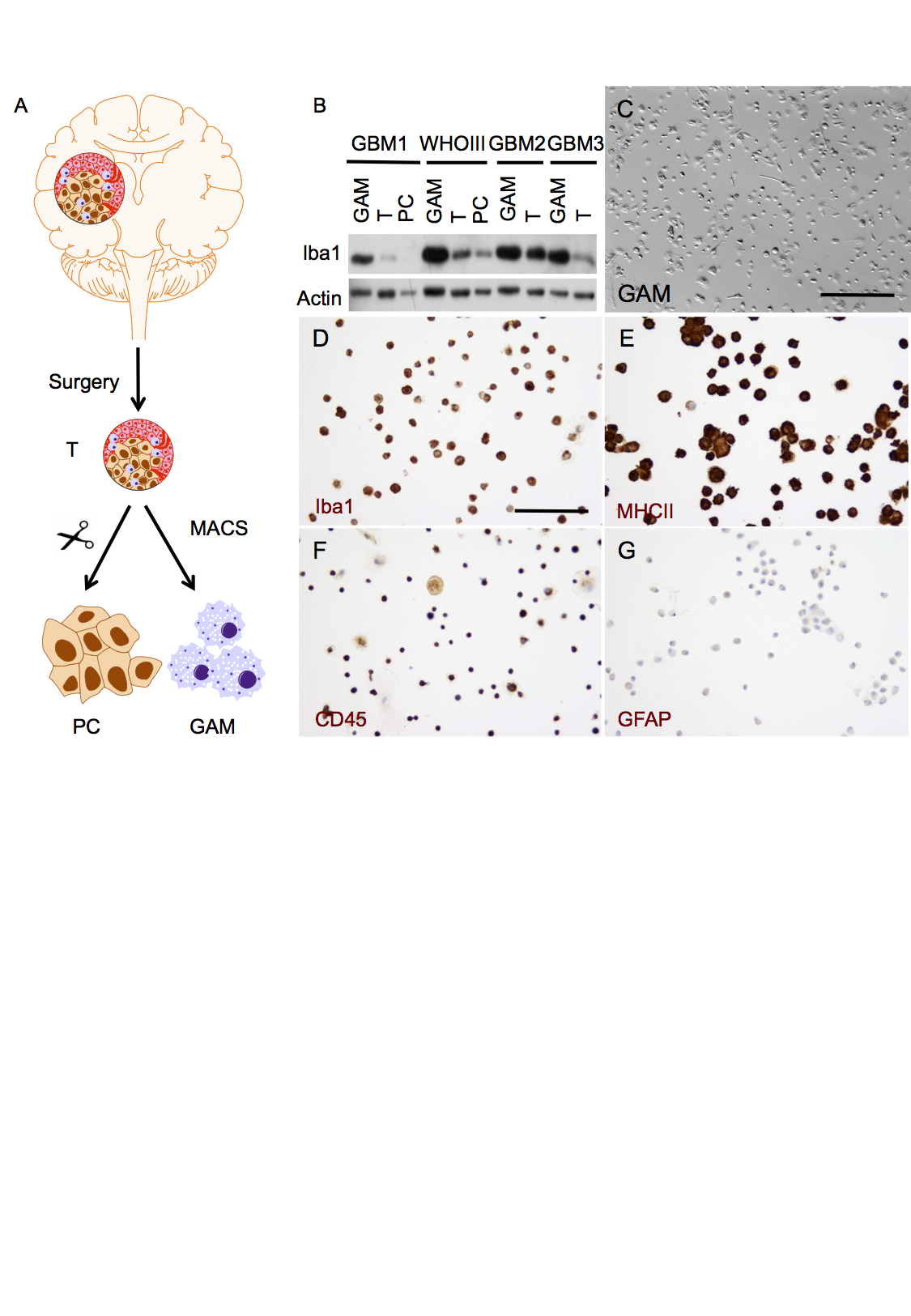

Supplement: Supplementary file 1 — Figure S1. CD11b‐MACS® selected GAMs show a high purity. A. GAMs and primary tumor cell cultures (PC) were extracted from fresh human glioma tissue via CD11b‐based MACS® or enzymatic and mechanical dissociation, respectively. B. In individual patients with high‐grade astrocytomas (GBM1‐3, astrocytoma WHO grade III), corresponding protein lysates of GAMs, PCs and the whole glioma tissue (T) were investigated for Iba1‐expression. Actin served as positive control. The CD11b‐MACS® selected GAM suspension C was analyzed by immunocytochemistry stainings for the classical M/M markers D. Iba1, E. MHCII, F. CD45 as well as (G) GFAP as a negative control and indicator for a potential contamination with tumor cells (original magnification b–h: 20×, scale bar = 100 µm). [file BPA-29-513-s005.tiff]

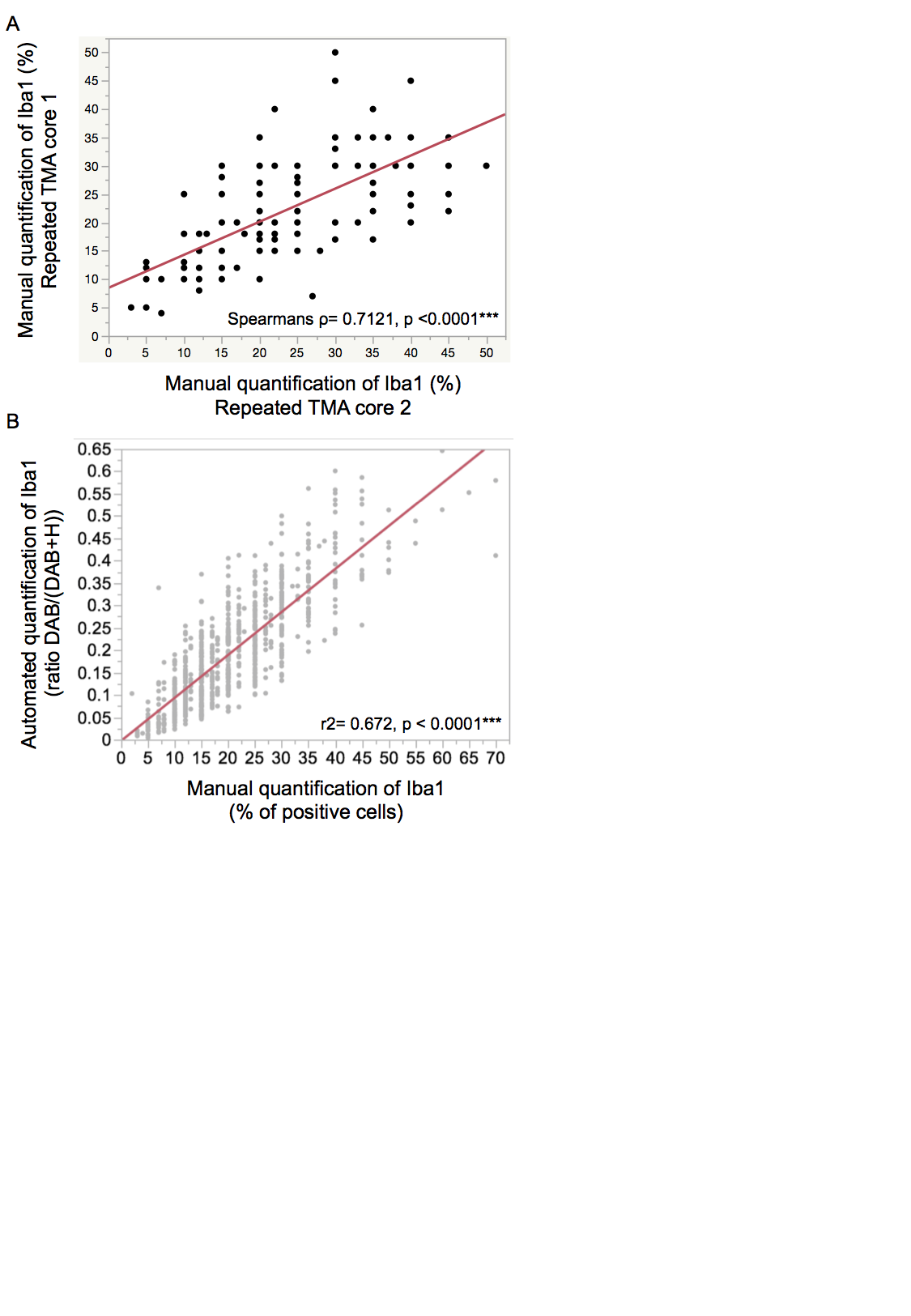

Supplement: Supplementary file 2 — Figure S2. Manual and automated quantification of Iba1‐immunohistochemistry. A. Correlation analysis revealing a high similarity of Iba1 levels between repeated tissue cores of individual patients of our TMA patients’ cohort (Spearmans ρ = 0.7121, P < 0.0001). B. Correlation analysis revealing that manual and automated quantification of Iba1‐immunohistochemistry strongly positively correlate in our TMA patients’ cohort (r2 = 0.672; P < 0.0001). [file BPA-29-513-s008.tiff]

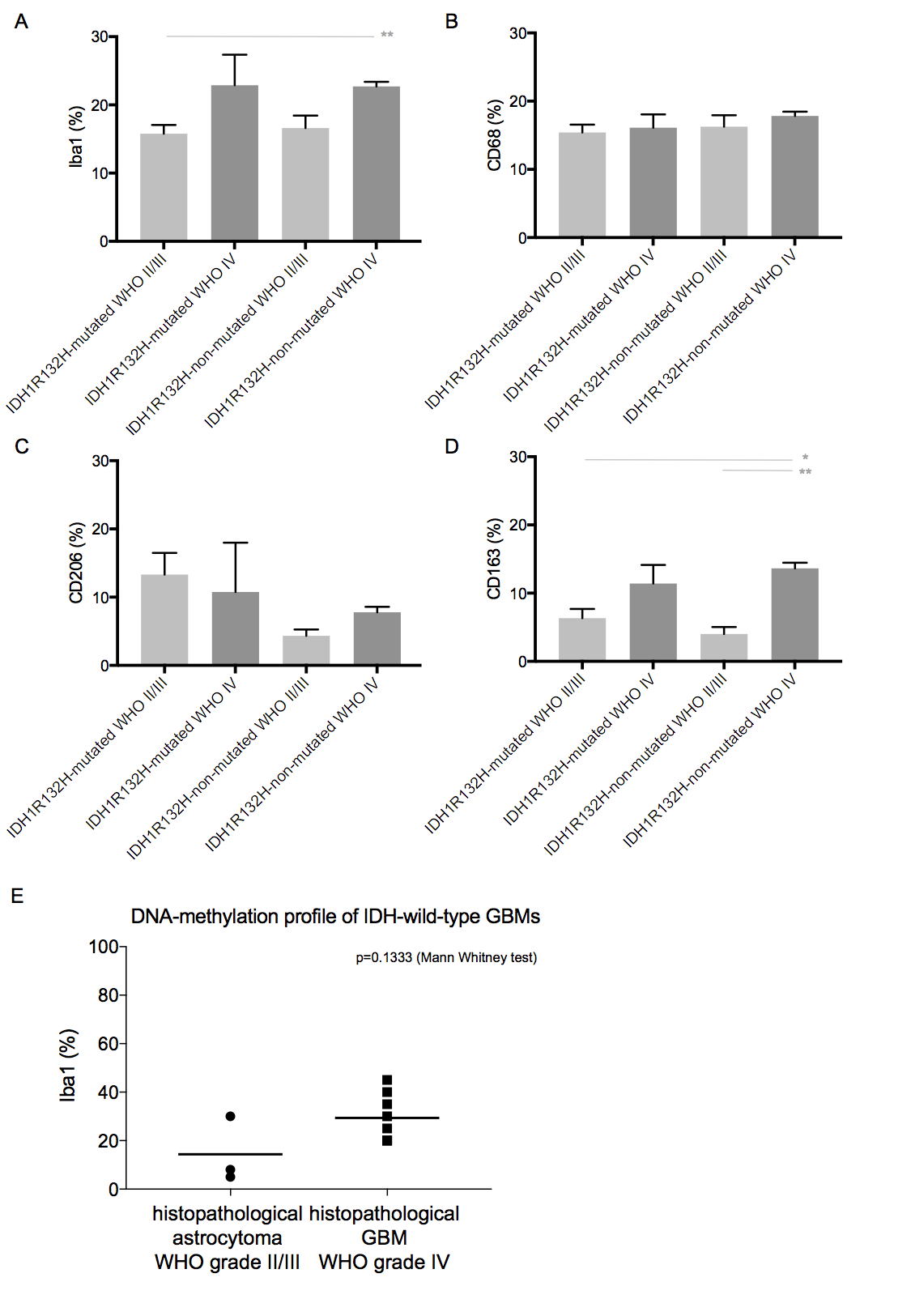

Supplement: Supplementary file 3 — Figure S3. Expression of Iba1‐positive GAMs in patients with diffuse lower grade astrocytomas compared to GBMs. A–D. Iba1, CD68, CD163 and CD206 levels were statistically assessed in IDH1R132H‐mutant and ‐non‐mutant lower grade astrocytomas (WHO II/III) compared to GBMs using the nonparametric Bonferroni's multiple comparisons test. Only significantly different expression levels between the different entities were highlighted. Statistical analysis was performed using GraphPad Prism 7 software. E. Iba1‐positive GAMs were quantified in methylation‐based classified IDH‐wild‐type GBMs with discordantly lower histopathological grading (n = 3) in comparison to patients with concordant classification of IDH‐wild‐type in both histopathology and methylation profiling (n = 8). Aligned dot plots for Iba1‐positive GAMs (in %) are depicted. The relative amount of positive cells was statistically assessed using the nonparametric Mann–Whitney test (P = 0.1333). [file BPA-29-513-s007.tiff]

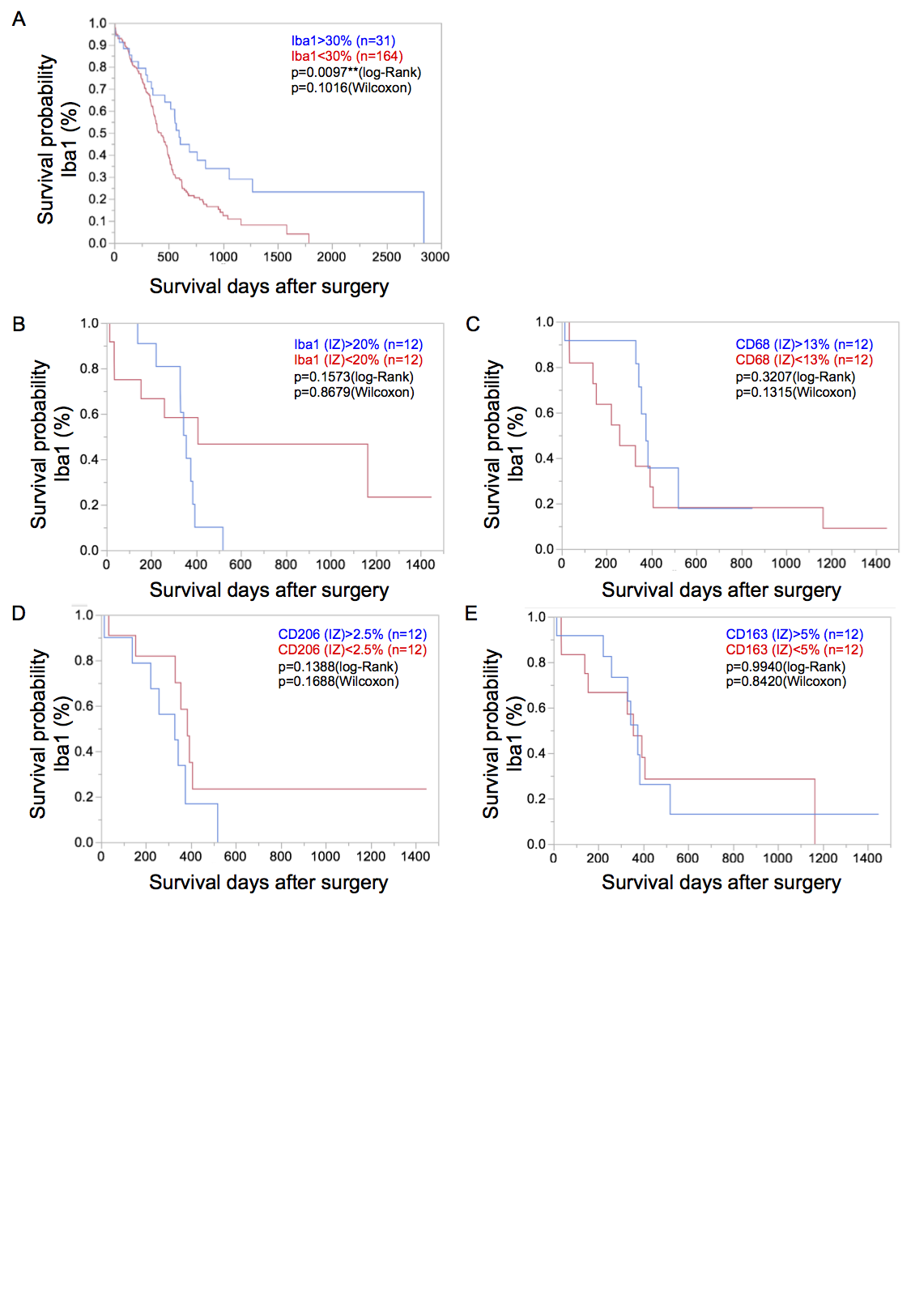

Supplement: Supplementary file 4 — Figure S4. High levels of Iba1‐positive GAMs in IDH1R132H‐non‐mutant GBM patients are associated with better survival. A. Kaplan–Meier survival curves of IDH1H132R‐non‐mutant GBM patients were obtained by performing best split (high expression >30% Iba1‐positive GAMs; low expression ≤30% Iba1‐positive GAMs) additionally to median split (20%, depicted in Figure 3B). B–D. Kaplan–Meier survival curves of Iba1‐, CD68‐, CD206‐ and CD163 positive GAMs in the infiltration zone (IZ) of GBM patients were obtained by performing median splits as indicated in the figure. Curves were compared by log–rank and Wilcoxon’s test (P‐values depicted). [file BPA-29-513-s009.tiff]

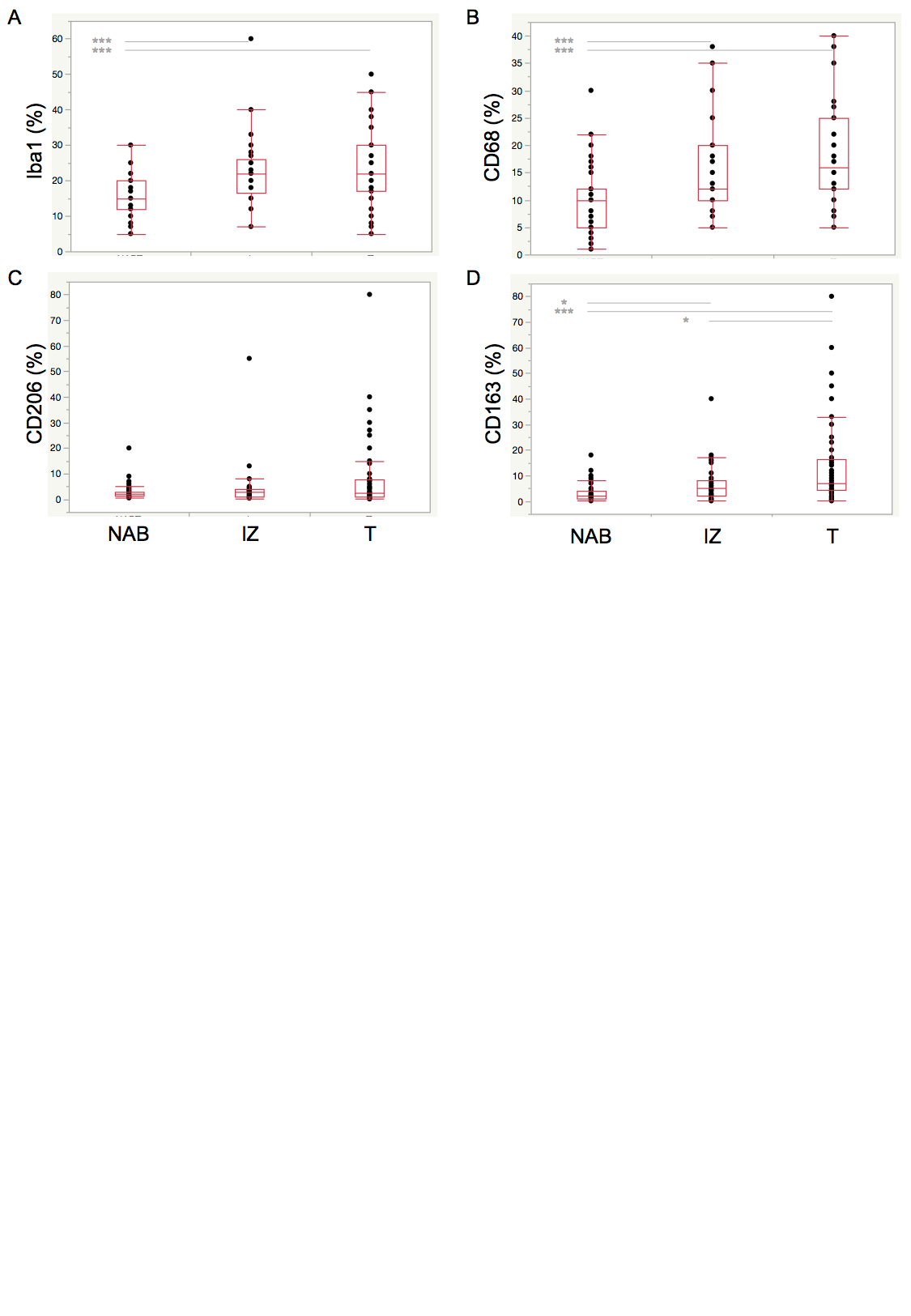

Supplement: Supplementary file 5 — Figure S5. Distribution of GAM subpopulations. This figure depicts the statistical analyses performed for the illustration of Figure 6. A. Iba1‐, B. CD68‐, C. CD206‐ and D. CD163‐positive GAMs were quantified by IHC in the vital tumor center (T), the infiltration zone (IZ) and the normal appearing brain tissue (NAB) of IDH1R132H‐non‐mutant GBMs. Box and Whisker plots for positive cells (in %) are depicted. P‐values were indicated (*P ≤ 0.05; **P ≤ 0.01; ***P ≤ 0.001) after performing nonparametric Dunn testing. Only significantly different expression levels between the localizations were depicted. Statistical analysis was performed using JMP 14.0 software (SAS). [file BPA-29-513-s001.tiff]

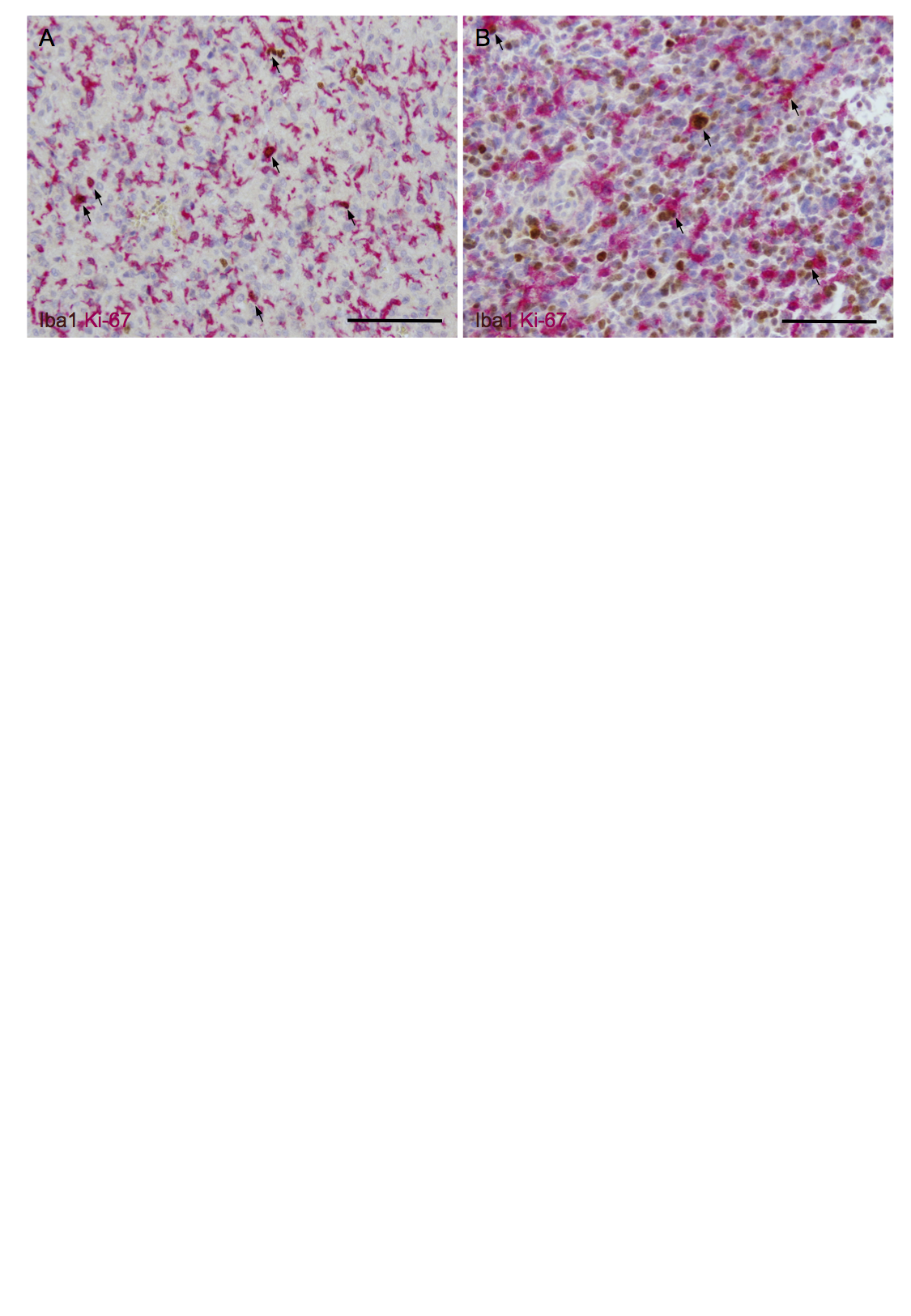

Supplement: Supplementary file 10 [file BPA-29-513-s006.tiff]
